# Design and feasibility testing of a novel group intervention for young women who binge drink in groups

**DOI:** 10.1371/journal.pone.0193434

**Published:** 2018-03-01

**Authors:** Linda Irvine, Iain K. Crombie, Vivien Swanson, Elena D. Dimova, Ambrose J. Melson, Tracey M. Fraser, Rosaline Barbour, Peter M. Rice, Sheila Allan

**Affiliations:** 1 Division of Population Health Sciences, School of Medicine, University of Dundee, Dundee, Scotland, United Kingdom; 2 Psychology Division, Faculty of Natural Sciences, University of Stirling, Stirling, Scotland, United Kingdom; 3 Faculty of Wellbeing, Education and Language Studies, The Open University, Milton Keynes, England, United Kingdom; 4 Division of Neuroscience, School of Medicine, University of Dundee, Dundee, Scotland, United Kingdom; 5 Dundee City Council, Dundee, Scotland, United Kingdom; Brown University, UNITED STATES

## Abstract

**Background:**

Young women frequently drink alcohol in groups and binge drinking within these natural drinking groups is common. This study describes the design of a theoretically and empirically based group intervention to reduce binge drinking among young women. It also evaluates their engagement with the intervention and the acceptability of the study methods.

**Methods:**

Friendship groups of women aged 18–35 years, who had two or more episodes of binge drinking (>6 UK units on one occasion; 48g of alcohol) in the previous 30 days, were recruited from the community. A face-to-face group intervention, based on the Health Action Process Approach, was delivered over three sessions. Components of the intervention were woven around fun activities, such as making alcohol free cocktails. Women were followed up four months after the intervention was delivered.

**Results:**

The target of 24 groups (comprising 97 women) was recruited. The common pattern of drinking was infrequent, heavy drinking (mean consumption on the heaviest drinking day was UK 18.1 units). Process evaluation revealed that the intervention was delivered with high fidelity and acceptability of the study methods was high. The women engaged positively with intervention components and made group decisions about cutting down. Twenty two groups set goals to reduce their drinking, and these were translated into action plans. Retention of individuals at follow up was 87%.

**Conclusions:**

This study successfully recruited groups of young women whose patterns of drinking place them at high risk of acute harm. This novel approach to delivering an alcohol intervention has potential to reduce binge drinking among young women. The high levels of engagement with key steps in the behavior change process suggests that the group intervention should be tested in a full randomised controlled trial.

## Introduction

Binge drinking among young women is a recognised public health problem [1]. Binge drinking, involves the consumption of more than six UK units of alcohol (48g of alcohol) on one occasion [2]. A common pattern for young women is having two to three occasions in a month, at which large quantities of alcohol are consumed. These episodes are interspersed with many alcohol-free days [3]. This type of drinking, also termed heavy episodic drinking, has been widely documented among young people [4]. A recent health survey in Scotland revealed that almost a quarter of women aged 16 to 34 years consumed more than six UK units of alcohol on their heaviest drinking day in the past week [5]. Binge drinking places young women at risk of violence, injuries, drink spiking and unintended and unprotected sex [6–8]. Drinking heavily in pregnancy poses serious risks to the fetus [9]. Women may consume harmful amounts of alcohol during the pre-pregnancy recognition period [10] and those who are regular binge drinkers are less likely to stop during pregnancy [11–13].

Reducing the alcohol consumed during binge drinking sessions is challenging as heavy drinking is viewed by many as a normal activity and being extremely drunk is socially acceptable [14]. Drinking sessions are often carefully planned and are viewed as an important part of young people’s lives. This has been described as calculated hedonism [15]. Young women frequently drink in social groups and binge drinking within these groups is common [16, 17]. Lange et al. have introduced the term ‘natural drinking group’ to describe small groups of people, ‘bonded by friendship or other interpersonal relationships’, who regularly drink together [18]. For young people, drinking is mainly about friendship and having fun, where the experience is shared rather than individual [19]. Much of the pleasure from drinking alcohol comes from developing and sustaining important relationships and bonding with friends [20].

Alcohol brief interventions (ABIs) are widely used to tackle problem drinking [21]. ABIs are a heterogeneous group of interventions which are delivered in single or multiple sessions. They can contain Motivational Interviewing, feedback and advice, self-monitoring of alcohol consumption, self-help manuals, counselling, and cognitive behavioral therapy [22, 23]. Many ABIs involve a single session with a total intervention time that can last from 7.5 to 60 minutes. Trials of ABIs with multiple sessions typically have total intervention times of 50 minutes or less [21]. Several systematic reviews have concluded that individual level ABIs are effective [21, 24]. However, the most recent systematic reviews report that these are unproven in women because of insufficient data [21, 22, 25]. Alcohol interventions have also been shown to be effective in college students although systematic reviews show that the effect sizes are often small [26, 27]. These interventions may be delivered college students individually or in a group setting [28]. A recent meta-analysis reported that those containing goal setting and expectancy challenges were associated with a larger reduction in alcohol consumption [28].

Reducing harmful drinking by young women is complex, because interventions have the potential to challenge relationships that are highly valued. Group norms can encourage heavy drinking [29, 30], but can also promote moderate drinking [31, 32]. Several authors have recommended that interventions to tackle binge drinking in young people should be targeted at natural drinking groups [16, 17, 31].

This study describes the design and assesses the acceptability of a community based, tailored intervention with pre-existing friendship groups to tackle binge drinking among young women. The novel intervention addresses drinking as a fundamental part of friendship, and seeks to highlight how fun and friendships can be maintained while reducing binge drinking. The objectives for the study were to develop and test the feasibility and acceptability all of the methods that will be required for a full randomised controlled trial. Feasibility will be measured by the success of recruitment, retention, measurement of outcomes, delivery of the intervention and engagement with the intervention. Acceptability will be assessed by a post-study evaluation of participants’ experiences of the study. The UK MRC guidelines on developing and evaluating complex interventions [33] stress that initial testing should take place prior to evaluation of effectiveness. Weaknesses in study design and methodology can be identified and addressed before undertaking a randomised controlled trial [34].

## Materials and methods

Ethical approval for the study was obtained from the University of Dundee Research Ethics Committee (Reference: UREC 12072). Women, aged 18–35 years, who regularly socialise and drink together, were invited to take part in the study with their friendship groups. For the study ‘friendship group’ was self-defined by the participants as a group who regularly drank together. The age range was chosen because women in this group have the highest prevalence of binge drinking among women in the UK [35]. Groups of 3 to 8 women were eligible if more than half of the group members had consumed more than six UK units (>48g of alcohol) in one session (binge drinking), on two or more occasions during the previous four weeks. This approach allowed the recruitment of those not meeting the binge drinking criterion so that friendships groups taking part in the study could remain intact. Retaining the integrity of pre-existing groups would maximise the impact of group processes on drinking behavior. All participants gave their written informed consent prior to recruitment.

### Recruitment strategy

Comprehensive co-ordinated recruitment strategies are recommended for community based studies [36, 37], including mass media approaches, community outreach and web based recruitment [38, 39]. Participants covering the spectrum from low to high deprivation were recruited. Socioeconomic status was assessed using the Scottish Index of Multiple Deprivation, an area based index which allocates scores based on six domains: current income, employment status, housing tenure, health, educational attainment, and access to communication [40]. Potential participants were purposively recruited in six ways: face to face recruitment by the study researchers in the community (shopping centres, high streets, community centres, cafes, gyms, student unions); advertising on two local radio stations; poster panels on buses; university and NHS intranets; a Facebook page; and a poster campaign. The study was advertised as a research project on women’s health and alcohol which focused on “looking good and feeling great”. The tagline *“Glitzed not Blitzed*: *a study of women and alcohol”*, was used to present the study as an opportunity to have fun with a serious purpose. All forms of advertising provided a landline phone number, a mobile phone number, an email address, a short code and a website address. Potential participants were invited to express an interest in the study by contacting the research team by any of these methods. An individual who expressed an interest in taking part in the study nominated the group of friends she regularly went drinking with and decided if they were eligible on the entry criterion. This spokesperson was interviewed by telephone to screen the group for eligibility. All group members were then sent a Participant Information Sheet, which described the study in detail.

### The intervention

The literature on young women’s drinking identified that pre-loading i.e. drinking in a private residence before going out to clubs or bars [41] and binge drinking [1] are common, being extremely drunk is socially acceptable [14] and that drinking is mainly about friendship and having fun [15, 20]. As this literature is limited to studies on individuals, focus groups were conducted to explore the nature of drinking in groups and to inform the intervention.

### The formative research

Seven focus groups involving 45 young women who were friends or work colleagues and who drank together regularly were used to provide information on the shared group experience. The women were from diverse socioeconomic groups including university students, young single mothers and mothers requiring social support, a netball team, solicitors, social work department employees and mothers attending playgroups. The discussions were recorded and transcribed verbatim and analysed using Framework Analysis [42], which was facilitated by NViVO 9 software. Transcripts were read and initially coded. Two researchers (TMF, AJM) then examined 4 transcripts for inter-coder verification of emerging codes and themes [43]. To check code interpretation, categories and enquiry lines, analysis meetings involving the whole research team were conducted.

The analysis identified four major themes: pre-loading, planning of drinking sessions, risk perception and motivation to change. Pre-loading, drinking before going out, was important for all groups. It often started as way to save money but then became an important part of the ritual of going out in a group. Group drinking sessions were usually carefully planned, but the frequency of these varied according to factors such as responsibilities and disposable income. Many women did not perceive alcohol-related risks as being relevant to them, despite being regular binge drinkers. Most had set their own perceived ‘safe limits’ for drinking which were gained through past experience.

None of the women were motivated to reduce their alcohol consumption. They felt that life circumstances e.g. family and work commitments, would dictate their alcohol consumption in the future so they should enjoy drinking while they can. The majority of women could not identify any social group activities that could replace binge drinking with friends. The perceived benefits of relieving stress, escapism and enjoyment were important to them. They also felt that peer influences would make it difficult to reduce their alcohol consumption. Even if they could resist peer influences, they could see few attractive alternatives to alcohol that would offer the same rewards and felt that there was nothing else available to do with their group of friends.

### Intervention development

The development of the intervention was underpinned by the principles of the Health Action Process Approach (HAPA) [44], a model emphasising the importance of motivational and volitional phases of the behavior change process. It was anticipated that motivation to change drinking behavior within the target population would vary, as would understanding and confidence in using strategies to enact and maintain changes to drinking behavior. The HAPA incorporates and specifies the relationship between these aspects of the behavior change process, whilst also serving as a useful framework for integrating additional components (e.g. subjective norms [45]). It also provided a logical sequencing for the behavior change techniques used in the intervention [46].

The intervention was delivered over three face-to-face sessions, approximately one week apart, to systematically lead participants through the behavior change strategy. Motivational Interviewing (MI) techniques, shown to be effective with groups [47] were used to explore and develop motivation for change as well as facilitating group discussions The specific techniques used were expressing empathy, using open questions, affirmation and reflective listening. These were used in discussions highlighting the discrepancy between desired outcomes of drinking sessions and consequences of excessive alcohol consumption [48, 49]. This was followed by a decisional balance exercise and the subsequent change talk provided the opportunity to gain commitment to change.

Each session was structured around a fun social activity in order to enhance engagement, lessen any concerns about research participation and act as a springboard for discussing intervention topics. Following the structure of the HAPA model, the first session was designed to promote motivation to change drinking behavior by encouraging the women to review alcohol outcome expectancies and assess their perception of risk from alcohol related harm. Group activities were designed to increase action self-efficacy and increase intention to change behavior. The second session addressed the motivational phase of HAPA by introducing goal setting and action planning, with activities to enhance both action self-efficacy and coping self-efficacy. The final session focused on maintenance of the changed behavior, an important part of HAPA, by addressing relapse, recovery self-efficacy and action control. Each session lasted between one and one and a half hours.

#### Session 1: Promoting motivation/intention to change

This session began with an ice-breaker, making and tasting alcohol free cocktails, which replicated the features of a drinking session but demonstrated having fun without alcohol. Using MI based techniques [50] the women were encouraged to explore the benefits and the adverse effects of getting drunk and the subjective importance of the drinking experience [51].

These discussions were followed by a decisional balance exercise [52]. The aim was to develop discrepancy between the anticipated outcomes of individual and group heavy drinking, such as having fun and group socialising, and the negatively valenced outcomes such as having a hangover or being unable to remember what happened the night before. This helped generate change talk about drinking, both at an individual and group level. The normative beliefs (perceptions of significant others’ beliefs) underpinning subjective norms (perceived social pressure to conform with others’ beliefs) were explored [45]. Exploration of intra-group variability in attitudes towards heavy drinking was intended to help overcome false consensus on high levels of alcohol consumption [53]. Action self-efficacy [44] was addressed through an exercise to assess confidence in being able to make changes to current drinking patterns.

#### Session 2: Setting goals, developing implementation intentions, and action plans

To develop the theme of looking good with confidence, a makeup demonstration was incorporated into this session. The group’s previous discussion about their drinking patterns was reviewed and the advantages of setting goals to achieve behavior change was introduced [54]. Guided discussion and information on goal setting was included to support translation of motivational readiness into specific goal intentions. The features of suitable goal selection were explained and the women were guided through the process of identifying and setting goals (SMART goals) [54], while the group discussions explored whether a suitable goal could be identified and negotiated at a group level. Identifying a group goal has the advantage that possible conflict between multiple individual goals operating within a single group context is avoided. The groups then devised action plans specifying ‘where’, ‘when’ and ‘how’ they would undertake specific actions to help them achieve their goal [44]. Action and maintenance self-efficacy encourage initiation and persistence of efforts to attain goals. Action self-efficacy was addressed by identifying barriers and facilitators to reduced drinking and discussing the importance of the group goal and confidence in attaining it. The importance of and confidence in enacting the group action plan was reviewed to strengthen maintenance self-efficacy.

#### Session 3: Coping plans, relapse prevention and habit formation

A demonstration of self-administered relaxation techniques provided an introduction to the session on coping planning and relapse prevention. The women discussed the goal they set in the previous session and were given feedback and encouragement on their attempts to implement it. Reflecting on and discussing experiences of goal pursuit and action plans was included to reinforce existing goals and plans and provide an opportunity to refine these where necessary [55].

The discussion then focused on relapse prevention and maintenance of reduced consumption by generating coping plans and using problem-solving techniques to maintain healthy levels of drinking. Risky situations, when binge drinking was likely to occur, were identified and discussed by group members who were encouraged to identify specific actions that could prevent a lapse from occurring (i.e. if x occurs then I will do y) [56].

Finally, to promote maintenance of changes in behavior, positive discussion was encouraged through focusing on successes (no matter how small). Discussions drew attention to desirable outcomes which had either been achieved or had the potential to be achieved e.g. weight loss, financial gain, fewer hangovers or improved family relationships. Women also discussed and identified possible rewards that would encourage maintenance of change.

#### Delivery of the intervention

The group interventions were delivered by trained lay peers. The lay peers were trained in informal small group sessions with an emphasis on practising the skills required to deliver the intervention. This was supported by a user-friendly manual which provided a step by step guide on all tasks to be performed. The training covered: questionnaire completion; introduction to techniques from Motivational Interviewing; decisional balance and change talk; goal setting, action planning, and habit formation. Role play formed an important part of the training. All of the intervention sessions were audio recorded and assessed for fidelity of delivery using a checklist. Feedback was given to the lay peers by a member of the research team (EDD) after every session.

### Baseline assessment

Baseline questionnaires were self-completed by the participants at the beginning of the first session. Responses to the questions were confidential and were not divulged to the group. Data collected included age, education attainment, marital status and employment status. The Timeline Follow Back (TLFB) [57] was used to measure alcohol consumption during the past 30 days. The TLFB interview is a well-validated and reliable self-report method used to measure alcohol consumption. The Fast Alcohol Screening Test (FAST) [58] was completed to assess the extent of hazardous and harmful drinking.

### Process evaluation

Engagement with the intervention was assessed by inspecting data generated during the three intervention sessions. Flip charts were used to record and summarise group decisions made during every session (e.g. decisional balance, goal setting and action planning). These charts were retained for use in subsequent sessions to ensure continuity across the three sessions. The data from the charts were transcribed, then grouped into themes and coded to provide frequencies of the decisions taken by the women.

### Follow up

Women were followed up four months after the intervention. Several techniques to promote retention were put in place [59]. The intervention sessions were organised to be convenient for participants, both in location and timing. Participants were given travel expenses and were entered into a prize draw on completion of the study. To maximise follow up, several methods of contact (mobile phone, postal address and email address) were obtained. Multiple attempts at contact were made during the follow up phase of the study. Where possible, groups of women returned for a face to face session to complete follow up questionnaires. When this was not possible, telephone interviews were conducted or the questionnaire was emailed or posted to the participants. Questions on alcohol consumption asked at baseline were repeated at follow up.

#### Assessment of acceptability of the study

Participants completed a post-study evaluation questionnaire to assess the acceptability of the study. Participants were asked whether they felt they had benefitted from taking part, whether they had discussed the study with anyone, apart from members of the study group and whether they would recommend the study to others. Participants were encouraged to given reasons for their responses to the yes/no questions.

## Results

### Recruitment

In total, 123 individuals contacted the study team in response to the advertising campaign. Of these 33 responded to the radio adverts, 8 to the poster panels on buses, 10 to the University intranet adverts, 10 to the Facebook page and 30 expressed an interest when approached by researchers on foot. A further 8 women heard about the study by word of mouth. One person contacted the researchers in response to seeing a poster. Finally, 23 women responded using an SMS short code (this method did not identify how individuals found out about the study). The study researcher (EDD) attempted to contact all of these individuals. From these, the target of 24 friendship groups of women were successfully recruited.

### Baseline characteristics

The 24 groups included 97 participants. The number of participants per group ranged from 2 to 8, with a median of 4 women in each group. One group did not meet the entry criterion of having the minimum number of three participants. At recruitment the group included three women, but one failed to attend the first session. The lay person who conducted the intervention session took a decision to proceed with the two women who did attend. It would have been unfair to turn the two women away. As part of the determining the feasibility of the study a decision was taken to keep this group in the study.

Women of different ages, marital status and socioeconomic status were recruited (Table 1). More than two thirds were under 25 years of age and almost 60% were in a relationship. The Scottish Index of Multiple Deprivation [40], was used to assess socio-economic status. The recruitment strategy achieved its aim of covering a spectrum of socio-economic status to increase generalisability. Although participants were recruited from all areas within the city of Dundee, Scotland, more were recruited from disadvantaged areas. Approximately half of the participants were college students which is similar to all women in the age group [60].

**Table 1:** Demographic characteristics of participants.

Factor		Number of participants N = 97
Participants’ age (years)		
	18–19	21
	20–24	45
	25–29	14
	≥ 30	12
	Age not given	3
Relationship status		
	Single	31
	In a relationship	59
	Relationship status not given	7
Scottish Index of Multiple Deprivation (SIMD) quintile		
	1 (most socially disadvantaged)	22
	2	26
	3	15
	4	21
	5	11
Employment status		
	Employed (full time)	17
	Employed (part time)	41
	Unemployed	35
	Other	4
Highest educational attainment		
	University degree (attained or currently studying for)	51
	Vocational qualification/further training	24
	High school	21
Current college students		55

#### Drinking patterns at baseline

The predominant pattern of alcohol consumption was of infrequent, but heavy, drinking sessions (Table 2). Mean alcohol consumption on the heaviest drinking day in the previous 30 days was 18.1 units (SD 11.9), but more than half of the women had 25 or more alcohol free days in this period. Mean consumption over the previous 30 days was 59 units of alcohol (SD 46.6). More than 75% of the women were classed as hazardous drinkers, as measured by FAST [58].

**Table 2:** Drinking at baseline.

Factor			Baseline N = 97
Alcohol consumption			Units (SD)
	Mean consumption in past 30 days		59.0 (46.6)
	Mean consumption on heaviest drinking day		18.1 (11.9)
Number of binge drinking days in previous 30 days (>6 units on one occasion)			Number of participants (%)
	<2 days		26 (26.8)
	2–3 days		29 (29.9)
	4–5 days		23 (23.7)
	6–7 days		10 (10.3)
	≥ 8 days		9 (9.3)
Total consumption in previous 30 days			
	0–24 units		25 (25.8)
	25–49 units		26 (26.8)
	50–99 units		31 (32.0)
	100–149 units		9 (9.3)
	≥ 150 units		6 (6.2)
Number of alcohol free days in the previous 30 days			
	<15 days		2 (2.1)
	15–19 days		6 (6.2)
	20–24 days		33 (34.0)
	25–30 days		56 (57.7)
Hazardous drinkers (Positive FAST)			74 (76.3)

### Engagement with intervention components

Fidelity of delivery of the intervention was assessed by reviewing the data from the flip charts which indicated that the women had understood and carried out key tasks in the behavior change process (S1 Table). For session 1, all of the groups identified pros and cons of being very drunk and discussed the advantages and disadvantages of reducing their drinking. However, on the final task, rating the importance of cutting down and confidence in their ability to cut down, nine groups did not record scores. Four tasks were assessed for session 2. More than 90% of groups set goals and made action plans to reduce alcohol consumption. All but one group went further and identified barriers to changing drinking behavior and facilitators to achieving their goal. Recording of the importance of and confidence in their ability to achieve goals was less well done, with 17 of the 24 groups achieving this task. Two groups failed to attend the final intervention session, but all of the groups who attended completed the tasks (identifying high risk drinking situations, how to cope in these situations and possible rewards for sticking to their goals. Receipt of the intervention was then assessed by analysing the content of the data on the relevant components of the intervention for each session.

#### Intention formation (session 1)

All but one of the 24 groups identified many more advantages than disadvantages of reducing drinking. Perceived benefits of reducing binge drinking included: losing weight; having fewer regrets after a night out; not waking up next to a stranger; having more energy the next day; and saving money. Disadvantages of reducing included the fear of having less fun on a night out or feeling isolated and self-conscious when not drinking as much as their friends.

#### Goal setting (session 2)

Twenty-two of the 24 groups set a group goal to reduce drinking. Most of the group goals involved drinking fewer shots or restricting pre-loading activities (Table 3). Importantly, the plans were detailed and specific to each group’s drinking patterns. Thus, while one group set a goal to avoid drinking shots completely, another group agreed to restrict the number of shots consumed. Techniques to reduce pre-loading included: measuring drinks instead of free-pouring; and planning to reduce the amount of alcohol brought to pre-loading sessions. Action plans on how to achieve these goals were negotiated between the friends. Participants wrote down when; where; how; and with whom they would change their drinking.

**Table 3:** Goals set by the groups to reduce drinking.

Group goals to reduce drinking	Number of groups*
Avoid or restrict the number of shots consumed on a night out	7
Monitor or restrict amount of alcohol consumed during pre-drinking sessions	7
Monitor or restrict amount of alcohol consumed when out drinking	4
Restrict money taken on nights out	2
Plan alcohol free nights out	2
Restrict amount of alcohol taken to house parties	1

*two groups did not set a group goal, one group set two group goals

#### Maintaining changes (session 3)

The women identified many high risk situations including special occasions and celebrations, stress, boredom, arguments and disappointments, and pressure from friends. The group members then negotiated how to cope in different situations. For example one group suggested *‘if celebrating*, *then we’ll keep in mind we want to remember the night; only take £20 out*, *leave bank card at home’*.

To encourage maintenance of reduced drinking, the groups discussed how to reward themselves when they managed to stick to their goals. They were encouraged to make separate lists for rewards that involved spending money (affordable by money saved) and those that did not cost money. Suggestions differed according to the composition of the group, but common ones were shopping for shoes and clothes, visits to the cinema or music gigs, meals out or holidays. Free rewards included movie nights at home, cooking together, walks and hikes and pamper nights.

### Retention of participants

Participants were followed up four months after the intervention was delivered. Questionnaires were returned from 84 of the 97 participants (87%). Only one complete group could not be contacted. Comparing the difference in consumption levels between baseline and follow up showed a reduction of 6.8 UK units (SD 45.0) in mean consumption during the thirty day period leading up to the interviews. The reduction in mean consumption on heaviest drinking day was 2.1 UK units (SD12.2).

### Post study evaluation

Participants were initially asked to give their main reason for taking part to assess the effectiveness of the marketing strategy (S2 Table). Thirty-one women said that the study sounded interesting and a further 18 mentioned a specific interest in alcohol; 30 thought that it sounded like fun, and 8 wanted to help out with a research study (some women gave more than one reason):

‘*It sounded like a fun way to spend a few hours when we were stressed about fourth year’* [at university]

‘I thought it was a good idea to help out for the study and also to think about how excessively we drink sometimes’

The majority of women gave positive responses to questions assessing the acceptability of study. Almost 70% of the participants (57 women) who were followed up reported that they had discussed the study with others (friends, colleagues and family members), while 76 (90%) said they would recommend the study to other women.

In response to a question on the perceived benefits of taking part in the study, 71 participants (85%) said they benefitted and gave reasons why. Thirty five women reported that the study had raised awareness about their alcohol consumption:

‘The sessions made me more aware of not only my own but my peers (especially girls) drinking habits. When we discussed what was involved before a night out in the “getting ready” process I was unaware of how much my friends and I actually drank before actually heading out’.

Another perceived benefit (for 13 women) was that they were more likely to reflect on their drinking after taking part in the study:

‘I feel that I have realised that limiting what I drink can be beneficial to how I feel about myself. If I don’t drink then I don’t have a horrendous hangover and that I feel I have more control’

Setting group goals and action plans were key components of the behavior change strategy. Sixteen women reported that this session had been particularly useful:

‘Hadn’t heard of SMART goals before. Easy to set big goals but hard to achieve. More appropriate to make small steps’.

Women were asked whether they had learned any useful techniques during the study. Many related tips on how to avoid becoming very drunk such as:

‘Saying no. Finding other ways to socialise and have fun. Thinking healthy. Taking more pride in myself.’

‘I’ve realised that it’s important to reward yourself for setting goals and achieving them, and doing something different like a mocktail class with friends can be really good fun’

The role of the friendship group was emphasised in the study evaluation. Women realised, that at the beginning of the study, they were not aware of what group members felt about drinking, and were unclear about their group’s motivation for drinking:

‘Interesting to hear everyone’s thoughts on drinking as it is not something that is usually discussed’

‘Being honest about our drinking habits and how it impacts on our lives’

Some women reported gaining confidence in being able to refuse drinks when they were with the group:

‘Being able to talk to my friends when I don’t want to drink and them accepting I’m monitoring how much I drink’

The support of the group was important, particularly in implementing goals and action plans:

‘Being part of a group aiming for the same goal makes it more attainable’

‘Definitely follow the goal we set. A lot easier to do when everyone you are out with has the same goal. A bit of peer pressure NOT to do shots now which I never thought would happen’

The perception of having fun endured to the end of the study, but many participants also reported that the experience had been useful and interesting, and in some cases, brought the group closer together:

‘I think combining the sessions with fun activities was definitely important as it encouraged us to find new ways of spending time together rather than drinking’

‘Benefited our friendship, discussed new activities to do together’

## Discussion

This study has demonstrated that friendship groups of young women who engage in heavy binge drinking can be recruited to an alcohol intervention study. The use of a novel method of delivery, fun activities that provide a platform for behavior change activities, may have encouraged participation. The intervention was successfully delivered with almost all groups of women setting group goals and making action plans to reduce their drinking. Process evaluation indicated high levels of engagement with components of the behavior change strategy and retention was high. The post study evaluation showed high acceptability of study methods, and highlighted the important role of the friendship group in reducing hazardous drinking.

This is the first study to report recruiting and intervening on natural drinking groups in an alcohol intervention. Alcohol interventions have been delivered to groups of female students, but these did not involve natural drinking groups [61, 62]. Participating as part of a friendship group appeared to reinforce feelings of trust and belonging, such that implementation intentions and SMART goals were agreed at a group level [56]. This may explain why participants freely discussed their heavy drinking and made group decisions to modify their drinking habits. They reported that working as a group helped in changing individuals’ drinking behavior. This confirms previous research showing that capitalising on support systems that exist within small groups could increase intervention effectiveness [31]. These findings suggest that group interventions may have great promise in tackling binge drinking by young women.

A strength of the study was the ability to monitor engagement with components of the behavior change strategy in real-time. The flip charts were used as part of the intervention delivery to record the pros and cons of being drunk, the benefits of moderated drinking, goal setting, action and coping planning, and maintenance strategies. These also provide unobtrusively collected real-time data on the participants’ responses to the intervention components. They demonstrated that the women successfully carried out key tasks in the behavior change process (e.g. implementation intentions [56] and monitoring goal progress [55]). This provides a powerful method of process evaluation, an approach recommended for studies of complex interventions [63].

A high follow-up rate (87%) was achieved, which is higher than many alcohol brief intervention trials [64]. It was not possible to bring all the groups together at follow up but the women were amenable to be interviewed by telephone or to complete and return the questionnaire. In a full randomised controlled trial it would helpful to obtain alternative contact details at baseline e.g. the telephone number or e-mail address of a parent or significant other. In addition, keeping in touch during the follow up period by occasional text messages could give an opportunity to request new address details and to maintain interest in the study.

A possible weakness of this study is that the time period over which the intervention was delivered. Study sessions were held approximately one week apart. Some groups reported that they had insufficient time to implement their plans to reduce binge drinking before returning for the third session. For the full trial the intervention should be held over a longer period, four to six weeks, with a longer period between session 2 (setting goals) and session 3 (behavior maintenance).

This feasibility study did not have a comparator group. Its purpose was to determine whether women could be recruited and retained in a novel group intervention study on alcohol, whether they would engage with the intervention and find the study methods acceptable, and whether the proposed outcome measures could be measured [65, 66]. Although a feasibility study should not attempt to estimate the effectiveness of the intervention, even if a control arm is present [65], there are other benefits of including one [67]. For example, the inclusion of a control group would have given an indication of retention in both groups in a full trial.

## Conclusions

This study has shown that targeting natural drinking groups of young women provides a unique opportunity to reduce binge drinking. The use of the fun-based activities aided recruitment and promoted engagement with the intervention. The intervention successfully prompted the young women to set goals and make action plans. This approach has great potential, particularly for groups who are unaware that they are drinking at hazardous levels. This intervention should be tested in a full randomised controlled trial.

## Supporting information

S1 Table Process evaluation (Flip chart data). (DOCX)

S2 Table Post study evaluation. (DOCX)

S3 Table Women & alcohol data (SPSS.sav). (SAV)
