# Supplementary material for: Design and feasibility testing of a novel group intervention for young women who binge drink in groups
Source: PLoS One. 2018 Mar 1;13(3):e0193434. doi: 10.1371/journal.pone.0193434 (PMC5832245; doi:10.1371/journal.pone.0193434)
Supplement: S1 Table — (DOCX) [file pone.0193434.s001.docx]

**S1 Table Process evaluation (flip chart data)**

During the intervention sessions, flip charts were used to record key discussion points during the intervention sessions:

**Session 1**

Pros and cons of being drunk

Advantages of not getting drunk during a drinking occasion

Disadvantages of not getting drunk during a drinking occasion

Importance of changing current drinking pattern

Confidence in ability to change alcohol consumption

**Session 2**

Set a SMART group alcohol-related goal

Barriers and facilitators to goal attainment

Importance of setting a goal

Confidence in achieving the goal

Formulate a group action plan to achieve the goal, including *when*, *where* and *how*

**Session 3**

Identify risky drinking situations (real or anticipated) i.e. when it is difficult to achieve the goal

Develop ‘if-then’ plans by identifying specific barriers and specific actions

Identify rewards as an alternative to alcohol that cost/don’t cost money

All data have been anonymised

**Group 0201, Session 1**

| **Pros and cons of being drunk**  **Pros**   - Getting dressed up - Fun - Lose inhibitions - Make new friends - Feels nice – euphoric - Have dance (pro dancer) - Feet hurt less - Don’t feel cold - Do silly stuff - Something to do - Cheap way to have fun - Spending time with friends - Reduces anxiety   **Cons**   - Having a bad night – obligated - Risk taking – dangerous situations - Morning after - Health related issues, short term + long term - Hangovers - Smoke more - Eat more, saying doing things wouldn’t normally do - 24hour takeaway - Weight gain - Never do anything next day - Productivity the next day - Making a ‘tit’ of yourself - Doing things wouldn’t normally do - Fights lashing out - Injuries - Not remembering things - Spending more once drunk - Not being able to drive |
| --- |

| **Advantages and disadvantages of not getting drunk during a drinking occasion**  **Advantages**   - Guilt free long lie - Save money - Driver – free drinks (union) - Lack of roughness - Productivity increases - Not so tired – waking not refreshed - Less toxins in body - Tidier - More time, feel better about life too - Long term –more confident - Less anxiety - No booze blues - Lose weight/not weight gain - More motivated - Skin better   **Disadvantages**   - Better value to buy in bulk - Easier to abstain than cut down - See friends less - Could spend more/same money - Meet less people/less social - Less to talk about/stories gossips - Missing special occasions |
| --- |

**Group 0201, Session 2**

| **Barriers and facilitators to goal attainment**  **Barriers**   - Other people buying them for you - People handing you them - Other people having them around you - Not feeling drunk enough - Everyone else drunk - Confidence – more carefree - Needing drinks to enjoy the venue - Practical element – not so many toilet trips |
| --- |
| **Risky situations**   - End of exams – blow out - Special occasions (birthdays, st paddys) – remind each other not drinking shots/buying - Deserve them – holidays – safety - Joining in with other people – having less at Pres using measure glass - Awkward situations – anxiety/new people - Not feeling happy/drunk enough, just going to pres - When you don’t want to go out - Who you’re with – not the group goal - On offer/freebies – the group ‘bombs’ slower, not shot them |
| Normal drinking night  10pm (vodka +juice before)  Pre-drink (approx. 2.5) – G+T, vodka + fruity diluting juice  12pm (travel with drinks)  Liar – Bar/club, shots jaeger bombs x 2 + drink/mingle straight away  Sofas, meeting new friends here  DJ  30min/1hour (stay in 2s of original group) some shots, drinks  Mono club – any mix. Go to bar as a group – some stay some go  Meeting point – shot + drink (prop) together  No drinks on dance floor.  Need for cool down, also go outside  Queue big  Rounds |
| **Facilitators**   - Doing it with friends - Not getting so drunk before – predrinking - Not taking out so much money - Taxi + couple of drinks + food - No bank card - Going to Air-nicer drinks - Thinking about the next day (productivity/activities planned) |
| **Goal:** No Shots  S – No shots in the Union, anywhere Liar, Mono, Air  M – count them  A –  R – G  T –  **When**: Fridays and Saturdays  **Where**: The Union  **How**: Group encouragement  **With**: each other  **Confident**: 8/9  **Important**: 7 |

**Group 0201, Session 3**

| - Reminding each other not to shot every time - Prinking – having less, different alcohol content (beer/cider), measures for spirits - Just going to ‘pres’ - Drinking bombs more slowly   Goal – no shots in the club  When: Any night  Where: anywhere/nightclubs  How: being aware of predrinks – how long there for/plan more  With: each other or 2 more |
| --- |
| **Rewards**  **Wee bit more cost:**   - Theatre trip - Out for dinner - DCA cinema club - Day trip – st Andrews, beach, get an ice cream - Having nice cocktails, 16^th^ May evening - Planning holiday - Hot air balloon – 2018 July - Café lunch - Afternoon tea   **Low cost:**   - Getting cat - Movie night - Pot luck dinner - Going for a walk - <name>’s cake - Having a bath – candles - Conditioning treatment at home, pampering night - Chatting to friends - Early night – book/dvd - Tidying room/fresh bed sheets - A nice healthy meal - Baking/cooking together |

**Group 0202, Session 1**

| **Pros and cons of being drunk**  **Pros:**   - Relax - Easier - Social scene - Masks the unpleasantness – smell, people, too noisy, people bumping into you - Seeing friends - Having a good time - Tastes nice – cocktails - Treat – justified/earned - Funny memories - Meeting people   **Cons:**   - Overly emotional during + post - Guilt - Post weight - Missing important things - Hangovers - Losing, dropping, breaking things - Cost – going out + holidaying away - Unattractive - False confidence - Smoking with drinking - Memory loss - Dangerous - Health – long term + short term - Bad skin - Bloating - Lose a day – fun things, need to do things - Working hungover - Facebook pictures |
| --- |
| **Advantages and disadvantages of not getting drunk during a drinking occasion**  **Advantages:**   - Save money * 5/6 - Better health – diet choices, not smoking reduces temptation *6/7 - Weight – calorific drinks - More productive - Respect – for yourself + for others - More in control – finance, body, mind, people around you *9 - Keeping the class   **Disadvantages:**   - Not seeing friends *10 - Some situations wouldn’t be fun - Not as much energy on the night - Night out not quite as fun - Explaining yourself why having less |

**Group 0202, Session 2**

| Pre-drinks – usually ready  8-9 pm. – spirits (3/4 drinks), champagne, wine. Home measures/free pouring. Usually finish bottles  9-10pm. – lift/taxi down:  -drinking different speeds  - starting off two groups of rounds  -end up buying own drinks  -spirits – 8  -shots (every 2^nd^ round): apple sourz (2), jaeggar boms (2-5), Sambuca (1 or less) (often do more shots when guys are out too – they buy them) |
| --- |
| **Goal:** Reducing pre-drinks  **Barriers:**   - Getting everyone together - Save money - Catch-up – easier - Drinks that you like |
| **Goal:** Measure your measures when pre-drinking – shot glass, on a <place> night out  **When**: <place> night  **Where:** each others’ houses  **How:** with shot glass + top up with mixer  **With:** each other |

**Group 0202, session 3**

| - Meet in <place name> pub – white wine spritzers - Being bored - Being in <name>’s house - Mecca Bingo - Aware of measures but not a focus - Stress – had a hard week - Meeting in a pub - Not starting off in someone’s house - The boys (influence) involved in night + buying drinks - Big night out planned - Non-planned night, start drinking and carried on - On a budget (drink loading) - Company influence |
| --- |
| - <Place>, not in your home – plastic shot glasses; measuring with caps; decanting into water bottle - Recognising the bar measures – what they are using - Counting actual drinks – using phone (app tally; drink selfies); holding onto glasses (something to symbolise the glasses-pennies, straws, post it notes) - Having quota - The boys – getting them involved - Bringing a limited supply of pre-drinks - Making one pitcher at the start - Not mixing spirits |
| **Rewards:**  **Pretty cheap:**   - Less of a hangover - Having a more productive day - A “free” day – sofa day, magazines, junk food - Movie night - Being a kid night – fart, board games   **Not so cheap:**   - Day out – getting nails done; zoo trip; go-carting - Shopping - Spa day – steam and sauna - Go out for a meal - Holidays   **Go out for a meal in April** |

**Group 0203, Session 1**

| **Pros and cons of being drunk**  **Pros:**   - Confidence – not so self-conscious - Giggly - Other drunk people - A laugh - Good memories - Celebrations - Social aspect - Meeting new people - Kopperberg beer gardens   **Cons:**   - Hangover - Weight gain - Looking after <name> - Working - Sore head/ill/dizzy - Brutal honesty - Money - Blackouts/memory loss – panic after - Dangerous/vulnerability - The shame (walk) - Too convincing acting (zombie) - How people treat you (them thinking you’re drunk + not), especially someone you care about - Other people not handling their drinks – your night’s ruined - Other drunk people - Overcrowded clubs - Injuries – to self and others |
| --- |
| **Advantages and disadvantages of not getting drunk during a drinking occasion**  **Advantages:**   - Weight loss/not putting weight on - More money - Less injuries - Feeling fresh - Gloating - More control   **Disadvantages:**   - Odd one out - Less tolerant of drunk people - Drinking too early on – night gets cut short |

**Group 0203, Session 2**

| 5/6 – have dinner, rose spritzers  6/7 – getting ready – wine – bottle between 2 (rose); coke/irn bru  7-9 – vodka red bull  9-11/11.30 – pub (couple) – vodka red bull x 4 (doubles); pints x 4; diet coke; shot with each drink (2-4-6): jagger bombs, bucky bombs, haggis bombs, skittle bombs. ; usually long drink + a shot  11.30/12 – 2.30/3 – union/underground/liquid; Union – still drink (cheap) –vodka lemonade + shot – 2-3 (6 measures); Double in tall glasses + 2 shots; 6-8 drinks – 32 measures; water |
| --- |
| **Group Goal:**   - Pre-drinkng – wait till finished getting ready - Shots – maximum shot total 4 “da” jaeger bombs - S – 1 in each pub; 1 in “da” club - M – 4 - A & R - √ - T – group motivation; wait till finished getting ready   **Goal** – 4 shots: 1 in each pub; 1 in the club  **When**: next night out  **Where**: pubs + union for shots. J’s house for pre-drinks  **How:** eyeliner, lipstick, drink selfie (per location)  **With**: J, A, K |
| **Confidence:** 6; 7; 9  **Importance:** 9 |

**Group 0204, Session 1**

| **Pros and cons of being drunk**  **Pros**   - Time to yourself - Relax/wind down - Enjoying with your meal - Socialising - Taste - More confidence - Feel good about yourself - Makes you happy - Cheap – depending on day   **Cons**   - Getting jumped - Having a drink and not being able to drive - Drinking around children - Hung-over – brain explosion - Falling about - Can’t say no - Cost – losing money - No remembering anything - Getting drink spiked - Pictures next day - Weight gain - Injury to self - Too much confidence - Head spinning - Forgetful - Dangerous things - Premature climbing objects - Aging - Bad decisions - Beer goggles - Going home with men - Retching in the morning - Waking up places up places you shouldn’t be - Vulnerable situations |
| --- |

| **Advantages and disadvantages of not getting drunk during a drinking occasion**  **Advantages**   - Have more fun - Spend quality time with children - Make better decisions - Use money for other things - Go expensive places - More energy - Weight management - Better health - Better skin   **Disadvantages**   - Drink less – wouldn’t be drinking - Feel the odd one out |
| --- |

**Group 0204, Session 2**

| Special occasions  Head to a venue – drink more, don’t dance, nightclub not really drink, bar/queuing  Measure what you’re drinking  Nights in:  2 glass wine  2/3 big WKD  Bulmer 3 bottles  Nights out:  5/6  Don’t know  2-3 fish bowls  10-12 bot WKD smaller/2/3 archers lemon  Bottle vodka, house party (70cl almost) – 2/3 vodka |
| --- |

**Group 0204, Session 3**

| **Risky situations**   - Heading out - Jaeger bombs –scare stories - Annoying/difficult company – make a plan, ignore them, take the car, find her a man, not, more to drink before, beg the bigger person - Stressed out – drunk a lot quicker (cook/tidy), drinking faster, distraction/purpose/therapy, walk away, time out in the bathroom, comfort eating - Caffeine tablets – drink less   Feeling self-conscious – drink more |
| --- |
| **Rewards**   - Going to the gym - Going for a nice meal - Face massage/massage - Retail therapy - Tidy - Sleeping |

**Group 0205, Session 1**

| **Pros and cons of being drunk**  **Pros**   - More confidence - More fun – pre drinks, parting at Dawns - Sociailising - Not caring - Happy - Something to do - Buzz - Getting dressed up - Cat walking in - Drunk photos/videos - Free drinks - ‘<name>s boys’ (knowing someone who’s out) - Seeing people you used to know - Guilt free junk food   **Cons**   - Having junk food, wanting to eat - Sweating – gets too hot - Sometimes boring - Other people judging - Blanking out - Falling, bruises – eyes, knees and head - Being sick - Getting chucked out - Arguments - Getting ditched – being on your own - Not getting in nightclubs - Regrets - Waking up in unusual house - Saying things you regret - Other people’s BO - Facebook pictures (bad pics) - Union photographer - Other drunk people - Creepy guys - Fag burns - Hangovers/all day hangovers - Breaking heels/ripped dresses - Wanting to drink again next day - Waste of a lot of money - Weight gain - Taxis |
| --- |

| **Advantages and disadvantages of not getting drunk during a drinking occasion**  **Advantages**   - Behave in a more responsible manner - Being able to walk to casino - Still have neat hair and makeup - Remember faces and names - Remember more – how you get home - Aware of what you are doing/actions - Less/no arguments - Over-reacting less - Not know when to stop   **Disadvantages**   - Feeling boring - Drunk people - Feeling sleepy when tipsy - Not speaking to as many people |
| --- |

**Group 0205, Session 2**

| 8pm:  All ready go for predrinking to Dawns  Vodka lemonade/coke  3/4/5/6 – tall glass. 4 fingers of vodka  10.30pm:  Bus/taxi to Union  What’s left, travel bottle 500ml  Get to the Union  2 x drinks and mixer  1 or 2 shots – bombs skittle/jaeger  20 drinks each + drink bought 4  3x3 slushies  250ml spirits – 125ml Morgan’s, 125ml vodka, ½ vodka between 2  After party at Casino |
| --- |
| **Goal** – drink a little less  S – when in the Union just buy 3 drinks  M – rather than 4  A –  R – Yes  T – Just buy 3 drinks each time  **When:** out in town – next time you’re out  **Where:** out in town/Dawn’s  **How:** count 3 glasses. Talk about it. Try to stick to the 3 drink goal. Help each.  **With:** Each other! |

**Group 0205, Session 3**

| **Challenges**   - Having money, have a limit, leave bank card at home e.g pay day – just buy 3 drinks when at the bar in the Union long drink 2 of your choice, taxi money (leave taxi money at home/taxi money to <name>) - Drinking more at Pre-drinks – buy a smaller bottle ½ instead of 70cl (35cl), not taking bottle in taxi, not mixing drinks - If someone else buys them – buy one less yourself - Had a bad day/week – think - The shot lady – think about the money, avoid them - Drinking inside – drinks with ice 3 – 1 longer 2 shots or long drinks - Just wanting to get drunk - Boring feeling left out – go out but use solutions - Buzz from music   Had an argument – hang out, make plans |
| --- |
| **£**   - Have a cake - Cinema - takeaway   **££**   - have a BBQ - food shop - zoo - camping   **£££**   - new trainers - holiday - tattoo - puppy - weekend away - clothes shopping - zoo - sky dive - TITP - Concert   After next night out – have a BBQ! In <place name> park (cook the sausages properly) |

**Group 0206, Session 1**

| **Pros and cons of being drunk**  **Pros**   - Socialising - Relaxing – drink to forget - Happy - Makes unfunny people funny - Dancing – silly moves - Feel (like) da boy - More confident – overall, get over awkies - Meet more people - Pictures – good + bad (funny) - Getting dressed up - Something to look forward too - Laugh at funny story   **Cons**   - Minor assault through dancing - Too confident - Over sharing - Too emosh - Regret of being more weird - Fighting - Losing things - Hangovers - Structural damage/property - Not productive the next day - Life dread - Forgetting everything – some blank bits - Blackouts - Apologising after - Falling over –bruises - Breaking ruining clothes/shoes - Spending money - Pictures - More drama - Unhealthy skin/diet |
| --- |

| **Advantages and disadvantages of not getting drunk during a drinking occasion**  **Advantages**   - Less severe/less regret - Less embarrassing things - Remember everything - Don’t need to apologise - Less severe life dread - Better memory of the night - More awareness - Better at handling confrontation - Save money   **Disadvantages**   - Looking after someone who is more drunk - Left out - More tired - Not enjoy night so much - Heels hurt - Miss happy/silly stage |
| --- |

**Group 0206, Session 2**

| The night  7-10pm Pre pres:  getting ready, just the girls  Wine 1-2 (not the smallest bottle) ½ bottle  Beer/cider 1 maybe 2 can/bottle 1-3  Vodka + limeaid, try to have 3 (trying to match pub measures)  10-12pm Pres:  Bigger group of people (new venue)  Wine finish the bottle  Finish total of 4 beers/cider  7-8 drinks – measures similar  4% tropical juice (carribean twist 70cl)  12.30/1pm, Venue of the dance floor ‘da club’:  <Place name>  2-3 shots of bombs  Skittlebombs 7/8  Sambucca  1 vodka mixer  Arrive – 3 bombs  <Place name>  0-1  1-2  Never shots  Vodka mixers  Maybe alcopops  Socialising – drinks as a prop  Depends on money |
| --- |
| **Goal:** Less hungover – more productive next day  Manage money better  In the club  S – setting a price limit (£5/10)  M - £5 member  A – Confidence  R – “  T – hangovers getting worse. Getting serious with degree. Setting a price limit of £5 in the club  **Where**: <Place name>  **When**: Next night out  **How**: Only take £5  **With:** each other!  Hang in bar  Enjoy the drink (taste) |

**Group 0206, Session 3**

| - Habit to have a drink – having a soft drink – water/soda water - Needing money at some point – having something before going out, limited portion at home, drinking milk/banana - Entry fee – fob/card, VIP, sports club, saving £1 coins and £5 - Borrowing money - Cash card – not taking it out, physical money – picking up money on way home, taxi+food+drink – trying to commit to walking - Peer pressure – have another drink/all friends having, talking about it, planning drinks, water - Satisfying – appropriate not over spending   Saving £1 for food |
| --- |
| **£**   - Sunbathe - Studying outside - Having a bath - Binge watching 4OD - Walking   **££**   - Long lunch - Drinks/chat - Trip to <place name> - Swimming - Cinema - Beach trip - Walking   **£££**   - Go on holiday again - Spa day - Weekend trip more local - Shopping - Day trip to <Place name>   26^th^ June trip to <Place name> for a long lunch |

**Group 0207, Session 1**

| **Pros and cons of being drunk**  **Pros**   - De-stress - Catching up - Feeling/buzz/on a high - Confidence - Lose inhibitions - Getting dressed up - Build up to a night - Guilt free eating - Taste - drinks themselves   **Cons**   - Drunk hiccups - Hangover - Smoke more - Confusing - Photos - Memory loss - Arguments - Other drunk people - Costs/wasting money on drinks out - Entry fee - Working hungover - Paying for taxis - Risky driving - Eating crap - Walk of shame - Spewing - Gambling - Violence - Falling - Breaking things - Spilling other people’s drink - Spilling your own drink |
| --- |
| **Advantages and disadvantages of not getting drunk during a drinking occasion**  **Advantages**   - Have a better night - Feel less bloated - Not so likely to be sick - Not as violent - Spend less money – spend money better - Better decisions – money related - Less one night stands - Feel better the next day - Still have friends/boyfriend/dignity the next day - Beer goggles   **Disadvantages**   - Annoyed at other people including friends - Feel a bit self-conscious - Sweaty people - Put a downer on your night - Feel a bit left out |

**Group 0207, session 2**

| To get a job  Specific – Handed out CV’s. Every Thursday after school  Measureable – Handed out 10 CVs, 2 hours  Getting call backs/rejections/offers/online applications  Attainable – 2 hours, 10 CVs  Job offer  Realistic –  Timely – Is this the right time for you to set this goal? |
| --- |
| 6pm – Vodka/Irn bru (starting off with small measure, 1 finger) ¼ bottle vodka  9pm – 2 x 3 finger vodka/irn bru  Taxi/lift to pick up <name>l  10pm –  11pm – Jugs of cocktails (Venmon, Woo Woos)  Share 1 between 2 (18-20 35ml vodka, ½ bottle)  4-5 jugs each  Vodkas, ½ bottle each  2 Kopperberg/Rose – 250ml (2 drinks)  11.30pm – 10 shots – Sours/Aftershock/Sambucca/Jaegerbombs  Underground/Union/Liquid/Out  Ameretto + coke  Vodka + coke (3-5)  Cocktails  Long vodka ¾ vodka  Sweeping drinks (0-2) 40 drinks  2am – Kebab shop |
| **Goal**   - Stop sweeping drink - In between drinks - Miss out the ‘inbetween drinks’, the Kopperberg/Rose   **When**: next night out  **Where**: <Place name> bar  **How:** Just not buying them  **With**: each other! |

**Group 0207, session 3**

| Missing out in between drinks   - Not really enjoying them - Wasting money - Mixing drinks - Getting more drunk to feel better stand other drunk people move - Peer pressure – commit to help each other - Other people buying drinks – take it slower, not buy replacement for the free drinks, share them, jug each, using a glass – use a straw - Drinks on offer - Money – leaving money in house, hiding money from self (taxi + food), not taken card out |
| --- |
| **£**   - Day trip - Water fights - Visit the beach - Pamper/spa day   **££**   - New bras - Makeup - New hair - Pamper self - Eyebrows/nails - Car - Paint balling - Spa trip - Tattoos - New piercings   Spa trip for the weekend in September 2014  **£££**   - Buy a yacht - Louise Viton - Car - Tattoos - Holiday - Buy a house - Pony - Private jet - Leon berger - Camel - Boob job - Plastic surgery - Personal trainer - Chef/dietician - New hair - Swim with sharks |

**Group 0208, Session 1**

| **Pros and cons of being drunk**  **Pros:**   - Social – getting together - Celebrating - Loosen up a little - Enjoying the night more - Dressing up nice – make-up - Build up – getting ready - More confident - Funnier - Music/dancing - Chatting up with old friends - Photos - Food   **Cons:**   - Expensive food next day - Making a fool of yourself - Speaking to people you shouldn’t be speaking to - Hangover - Photos - Throwing up - Tears – over emotional - Feeling obliged to go out - Regret – everything/anything - Crossing the line - Falling over - Breaking things - Taxi rides - Spinning toilet – cubicle - Other drunk people - Making fake friendships - Making up - Make-up everywhere - Too hot, uncomfortable - Blisters - Sweaty strangers |
| --- |
| **Advantages and disadvantages of not getting drunk during a drinking occasion**  **Advantages:**   - Make-up staying in place - Remembering things - Not as bruised – not falling - Less of a hangover - Not over emotional/arguments - Less likely to make a fool of yourself - Spending less money or losing your money   **Disadvantages:**   - Less confident - Feel left out |

**Group 8, session 2**

| 8:30 – 9 ish – prinks (pre-drinks)  At a house – WKD 1-2 bigger bottles; wine – 1 bottle  11.30 – taxi to Union (no taxi drinks)  To liar – cocktails, vodka based (small pint glass) 2/3^rd^  Mono – bombs – 2-5  12.30 onwards – Vodka lemonade 3; shots – 3: sourz/Sambuca/tequila  2/2.30 – food! |
| --- |
| **Goal:** Pre-drinks – share a bottle of wine between 2 people  Shots – stick to 4 shots or less (incl. bombs + shot shots)  **How**: take a picture; not buy each other; cashing shots in on different floors; shot blocking  **Where**: <Place name>  **When**: Next night out  **With:** Each other |

**Group 0208, Session 3**

| Pre-drinks – sharing a bottle of wine between 2 people   - No one else drinking wine – buy a smaller bottle - ½ bottle not lasting the pre-drinks timeframe – slowing down-smaller glass; leaving bottle further away; making up tall drinks; waiting 10 mins between drinks; use ice; not using a straw - Drinking games – finish drink then have time out; sneakily use soft drinks in the game - Emotional/upset ‘cause a bad day – get some food + bitch over chicken! - Peer pressure – avoiding (night out) – club/busy places; doing things in smaller groups; keep your hands full; pretend drinking the shots (use the ice); act drunk - Want to get drunk/ let go, why not – think about money; a good night doesn’t have to end in the casino; not getting involved in big rounds (sticking to 2s) - Special occasions – keep your hands full; sticking to the same drink; all of the above |
| --- |
| Stick to 4 shots or less  -take a picture  -not buy each  -cashing shots in on a different floor  -shot blocking   - Special occasions: peer pressure; not buying for each other; pick a lower % shot (sourz); hands full; act drunk/happy - Bumping into friends – choosing a weaker shot; just say no – whit a smile - Randoms buying shots for you – just give it away; ask for a long drink; say something to put them off buying it for you; spill it (on them) - Cheap shots (union) – 1 at a time; pause between drinks; be aware of how many you’re buying yourself; good selfish – recognise when you’ve had enough |
| **£**   - Night in – pamper night - Go for a walk - Day out - Cinema - Out for tea   **££**   - Shopping - Pampering yourselves - Nails done   **£££**   - Travel - Holiday - Nice car   Trip through to <Place name> for a girly night – near the 5^th^ of August 2014! |

**Group 0101, session 1**

| **Pro:** Social  **Con:** Cringey |
| --- |
| **Advantage:** Saving money  **Disadvantage:** Miss out on social events |

**Group 0101, session 2**

| **Goal:** Not drinking at casino  **How**: Avoid bar – distract: food + play games; stay together; encourage each other; get nice soft drink  **Where**: casino <Place name>  **When**: Saturday  **Who with**: Together |
| --- |
| **Barriers and facilitators to goal attainment**  **Barriers:**  Friends outside group, boredom, extra money, routine/habit, others buy you a drink, temptation  **Facilitators:**  <name>’s good influence, groups encouragement, money for other things, feeling better/free time |
| **Importance:**  4;  **Confidence:**  5;8;9 |

**Group 0101, session 3**

| **High-risk situations:**   1. People from outside the group 2. Plan on staying in but hear of friends going out 3. Special occasions 4. Tired from work 5. Family arguments 6. Special offers – <Place name>’s January sale – 20% 7. <name>’s dad 8. Bored 9. Too sober   **If this situation arises, I will…**   1. Go to the bar with group you are with, not new group 2. Find friends that are not going out: have a night in or go to the cinema 3. Take the car, have soft drinks, pretend you are pregnant 4. Sleep/ distract yourself 5. Going for a walk, visit friends/gran, take dog out instead of pub 6. Limit to a number of shots 7. Ask to have food instead 8. Take up a new hobby, put musing loud and clean room; go for food 9. Look after drunk friends, go home |
| --- |
| **Rewards that are free:**  Spend time with family  Better sleep/feel better next morning  Massage each other  Pamper night  **Rewards that cost money:**  Shopping  Go for food  Road trip  Buy cookies  Haircut/get nails done |

**Group 0102, session 1**

| **Pros and cons of being drunk**  **Pros:**   - Fun - Seeing friends/different groups - Break from uni work - Card games/drinking games are fun - Nice tastes/trying new drinks – treat - Chance to dress up - Chance to dance – less awkward - Meeting new people   **Cons:**   - Falling over - Hangovers - Silly arguments - Expensive - Getting emotional - Forgetting things/embarrassing - Falling asleep in public places: toilets |
| --- |
| **Advantages and disadvantages of not getting drunk during a drinking occasion**  **Advantages:**   - Losing weight - Feeling healthier - Spend less money - Eat less food after night out, next morning/day – always junk food - Better photos next day - Less hungover by not mixing drinks - No cancelling plans next day/ more productive - Less bloated   **Disadvantages:**   - Going home early – too sober - Less tolerant to drunk strangers - Feel pain in feet/be more cold - More socially awkward |
| **Importance:**  General: I: 3, R & A: 5, J: 6  Health: I, A & J: 8, R: 9  **Confidence:** 6;7;9 |

**Group 0103, session 2**

| **Specific goal:** Use actual measures when drinking at home  **Action plan:**  **How:** Use measuring cups, smaller glasses, leave everything in the kitchen so it is more effort to pour another drink, don’t let others pour the drinks outside the grouo  **Where:** Amber/Iona/Rachael’s flat  **When**: May sometime  **With whom**: Together  **How important**: I, A & J: 7; R: 6/7  **How confident**: R & A: 8, I: 10, Jo:8/9 |
| --- |
| **Barriers and facilitators to goal attainment**  **Barriers/unhelpful things:**   - Somebody else pouring it - Not appropriate glasses – too much mixer - Pressure to catch up with people outside the group - Drinking games - Feeling stressed – uni   **Facilitators/helpful things:**   - Having appropriate measuring tools - Supporting each other - Having set plans the next day |

**Group 0102, session 3**

| **Risky situations – How to cope with these**   - Not been out in a while – start drinking later - Final hand in ever at uni – celebration – inevitable - Results day – go for dinner, bring some money - Flat crawl- same amount of spirit in each drink - Other people buying drinks – pass on to somebody else - Friends haven’t seen in ages – limit how much money you take. Suggest other activities   Boredom – go for a walk, cinema, run etc.. going to <Place name> |
| --- |
| **Rewards:**  C**ost money:**   - Shopping - Cinema - Dinner - Day out in <Place name> - Go on holiday - Music festival   **Don’t cost money:**   - Go to the beach - Go for a walk - Movie nights in - Baking/cooking - Cleaning - Paint nails - Board games - Socialising – nowhere to rush - Gym |

**Group 0103, session 1**

| **Pros and cons of being drunk**  **Pros:**   - Sociable - Something to do - Reward/treat - Happier   **Cons:**   - Hangover - Dangerous - Aggressive behaviour - Embarrassment - Feeling on top of the world - Long term health consequences |
| --- |
| **Advantages and disadvantages of not getting drunk during a drinking occasion**  **Advantages:**   - Save money - Better liver - Better skin - Lose weight - Feeling fresh next day - Not waking up to strangers   **Disadvantages:**   - No buzz - Less tolerant of other drunk people - Feel more boring |
| **Importance:** 1, 4  **Confidence:** 3, 7 |

**Group 0103, session 2**

| **Goal:** No shots next night out  **How:** avoid shot girls, together to bar, tell people not to provide shots  **Where:** Union  **When**: 2 weeks, next time  **Whom**: together |
| --- |
| **Barriers and facilitators to goal attainment**  **Barriers:**   - Too sober - Being too drunk - Meeting friends outside group - Cheap shots - Sambuca tastes good - Shot girls walking about, easy access to drink, no queue - Buy more of other drinks   **Facilitators:**   - Reminding each other - Feeling better next morning - Some shots expensive |
| **How important to achieve goal:** 5,8  **How confident are you to achieve this goal:** 5 |

**Group 0104, session 1**

| **Pros and cons of being drunk**  **Pros:**   - Makes you happy/giggly - Relaxed/carefree - More in the present - Sociable - Feel more confident- looking better - Dancing/music - Make new friends - Tastes nice - Something to do – activity - Goes well with food + friends - Celebration - Funny stories   **Cons:**   - Injuries - Hangovers - Embarrassment - Sickness during night out - Sometimes makes you feel worse - Sore body next day/stomach - Doing silly things/regret - Blanks - Dangerous situations – walking home alone - Lose important things - Expensive - Can’t drive - Missing out on things the next day – disappointment - Doing things you regret - Calories – binge drink next day |
| --- |
| **Advantages and disadvantages of not getting drunk during a drinking occasion**  **Advantages:**   - General health benefits - Save money - More productive - Less injury - Less tired - Less likely to end up in dangerous/risky situations   **Disadvantages:**   - Less fun on nights out - Left out - No prep - Less tolerant to other drunk people - Less funny stories/moments - Make less friends-less interaction with other people |
| **Importance:** 1, 1,2,2,4,4  **Confidence:** 3,4,5,8, 10, 10 |

**Group 0104, session 2**

| **Goal**: No shots throughout the night  **How:**   - Don’t order them - Order something else - Inform others at pre drinks - Practise saying “no” - Support A - Order together - Whoever fails needs to buy everyone drinks after the night out   **Where:** <Place name>  **When:** Friday, 23rd  **Who with:** the group |
| --- |
| **Barriers and facilitators to goal attainment**  **Barriers:**   - Other people buying shots - Temptation/too sober - Quick fix - Others not understanding - Challenge to see if you will drink a shot - Shots are cheaper than other drinks - Already being drunk – harder to follow goal   **Facilitators:**   - Being in a good mood - Standing together - Knowing you are saving money - Taking out a certain amount - Dancing – doing other things and not going to the bar - Treating yourself to a nicer drink |
| **Importance:** 4,4,5,7  **Confidence:** 7,9,9,9 |

**Group 0104, session 3**

| **Risky situations: If – Then**   - Close friends outside group drinking shots – go to the bar without them - If too drunk – support from group/keep card somewhere (purse) - Too quiet/not enough distractions – could go home/change venue - Not been out in a while – being aware/drink more water, eat big meal before - Start drinking too early – buy less alcoholic drinks/pace yourself - Didn’t eat enough before – buy food at venue |
| --- |
| **Rewards:**  **Cost money:**   - Have ice cream - Go on holiday - BBQs - <Place name> - Road trips - Shopping - Go out for a meal   **Don’t cost money**   - Cook together - Go for a walk - Pamper night - Movie night - Board games |

**Group 0301, Session 1**

| **Pros and cons of being drunk**  **Pros:**   - Fun - Getting dressed up - Confidence - Spending time away from kids - Relax - Socialising - Less boundaries - Take aways   **Cons:**   - Hangovers - Spending lots of money - Make a fool of yourself - Time away from kids - Waking up for the kids during the night - Facebook photos - Drunk texts - Less boundaries |
| --- |
| **Advantages and disadvantages of not getting drunk during a drinking occasion**  **Advantages:**   - Less money - Not being sick - Less drinking - Less embarrassment - Less bruises - Listen to people   **Disadvantages:**   - Less drinking – drunker people are annoying - Listen to people |
| **Importance:** 2  **Confidence:** 8 |

**Group 0301, session 2**

| **Stop smoking -> have only five a day -> don’t smoke when bored** |
| --- |
| **Lose weight**  **S –** don’t eat after 8 pm; cut out junk food; eat breakfast; snack healthily  Don’t eat junk food throughout the week  **M –** you don’t have anything during the week |
| **How to avoid a hangover**  **S –** drink water in between drinks  **M**- by getting a glass of water between drinks  **A**  **R**  **T** – by the end of the night |
| **Barriers and facilitators to goal attainment**  **Barriers:**   - Kids - Going to the toilet - Waiting for a glass of water - Making the most out of it   **Facilitators:**   - Emotional support - Making sure you are prepared - Bottle of water in bag - Order glass of water with alcohol - Saving money |

**Group 0301, session 3**

| **Risky situations**   - Wedding - Break ups - Birthdays - Christenings - Death - First night out after having a baby - Exams - Graduation - Going out - New people - New Year |
| --- |
| **If at a party, then…**   - Have something to eat before - Drink water before bed - Painkillers - Don’t drink spirits - Take less money |
| **Rewards:**  **Free:**   - Long lie - Make time for yourself - Going out to town by yourself - Bath with candles and bubbles - Soap night – bed to yourself - Big brother night   **Cost:**   - Shoes - Clothes - Chocolate - Take away - Going out - Girls night - Cocktails - Movies marathon |

**Group 0302, Session 1**

| **Pros and cons of being drunk**  **Pros:**   - Confidence - Funny/more of a laugh - Good memories - Less boundaries - Drunken relations - Food - Sociable - Better singer/dancer   **Cons:**   - Stealing - Less boundaries - Hangover - Embarrassment - Facebook photos - Drunken one-night stands - Food – take aways - Injuries - Bad judgment - Hyperthermia |
| --- |
| **Advantages and disadvantages of not getting drunk during a drinking occasion**  **Advantages:**   - Less hangover - Weight loss - General health - Save money - Less embarrassment - More boundaries - Less injuries - Change in friendships (drinking buddies)   **Disadvantages:**   - Peer pressure - Not as social - Fussy - Other drunker people - More boundaries - Changes in friendships (drinking buddies) |
| **Confidence:** 8  **Importance:** 3 |

**Group 0302, session 2**

| Pre- 6.30 pm:   - Cocktail bowls - Shots - Wine - Test tubes - Spirits – vodka, gin - Beer, cider - 1 bottle of spirit - Travel bottles – maybe?   Club - 11pm   - Not a lot - If cheap will buy more - If busy will buy more - Buy more than one drink (at least 2-4) - Spirits, shots – cheaper   Casino/Flat   - What’s left over - Cider - Beer - Spirits - Shots   OR going home |
| --- |
| **Goal:** Not to have test tube shots  **When:** when pre-drinking  **Where**: flat  **How**: just don’t buy them  **With**: each other |
| **Barriers and facilitators to goal attainment**  **Barriers:**   - If someone bought them - If they were on sale - If drunk - Cocktails   **Facilitators:**   - If you weren’t in home bargains |
| **Importance:** 2  **Confidence:** 10 |

**Group 0302, session 3**

| **Risky situations:**   - Special occasions - Saturdays - Bad days - Good days - Sunny days - Work - Peer pressure - Exam stress - Celebrating end of uni/work - Holidays - Break ups - Onions - Birthdays - First class trains |
| --- |
| **If:** Sunny days**, Then** have a glass of water between drinks  **If**: Special occasions, **Then** drive/glass of water between drinks/group supports  **If**: Holiday, **Then** have a mocktail, keep busy with activity  **If**: Birthday, **Then** ask people not to buy drink as a pressie/buy singles not doubles  **If**: Work, **Then** have a rant/have a bath |
| **Rewards:**  **Cost:**   - Shopping - Handbags - Glass of wine - Get nails done - Pamper - Food - Bike ride - Music - Holiday - Mags   **Non-cost:**   - Bath - Paint our nails - Pamper - Food - Gigs - Movie nights - Sex - Reading - TV |

**Group 0401, Session 1**

| **Pros and cons of being drunk**  **Pros**   - Social - Confidence - Happy - De-stress- forget about stuff - Loss of inhibitions - Awesome dancer - Immense patter - Celebrate - Allowed to be self - No responsibilities   **Cons**   - Expensive - Sick - Spinning room - Hangovers – sore head; eating junk food; calories; not 100%; lose a day - Drinking munchies - Making a fool of self - Disappearing - Risky decisions - Safety - More at risk |
| --- |
| **Advantages and disadvantages of not getting drunk during a drinking occasion**  **Advantages**   - More money - Weight loss - No hangovers - Health benefits: liver, kidney, brain - Remember more of the night - No “beer” fear - No “beer” goggles - More varied social life   **Disadvantages**   - Loss of friends - You find people more annoying - Less tolerant - Not as much fun - May not be out whole night - Less confidence - Isolated |
| **Importance:**  Now: 1, 1, 2, 5  Near future: 1, 9, 6, 9  **Confidence:**  Now: 9, 0, 0, 3 (no motivation)  Near future: 9, 10, 9, 10 |

**Group 0401, session 2**

| **Barriers and facilitators to goal attainment**  **Barriers:**   - Mood - ↓ mood ↑drinking; extremes - Bad day at work - Social media - Friday feeling - Run out too soon   **Facilitators:**   - Working as group/social - Keeping you together |
| --- |
| ***Drink more**  ***Drink less go out**    **V:**  7-8: Chat/socialise; varies. 25ml. cap of alcohol plus mixer – 1/3 70 cl  8-9: Garden/smoking; 15ml. spirit mixer  9-onwards – half hour -> spirit mixer  **C:**  7-8 – house; wine 1 bottle or 3 ciders – 200 ml glass  10 – Jack Daniels hipflask – 1-2 finger -> night  *bring together  **S:**  1 bottle wine  2/3 Cava/4 cans less pint  ½ bottle gin |
| **Reduce alcohol consumption:**  S: 1. Drinking together in rounds 2. ½ bottle of spirit/1 bottle of wine  M: ½ bottle spirit (measure is a cap) / 1 bottle  A: √  R:  T:  **Confidence:** 10, 8, 8  **Importance:** 3, 9, 3 |
| **Goal:** Drink in rounds. ½ spirit or 1 bottle wine  **Where:** house party  **When:** get togethers  **How:** only bring a set amount: ½ spirit 1 bottle using cap as a measure  **With:** four of you |

**Group 0401, session 3**

| **Goal:** Be more drink aware  **When:** house parties  **Where:** -\|\|-  **How:** drinking in rounds; after every 3 drinks to see how feeling  **With:** group |
| --- |
| **In pub:**  **Barriers:** less likely to be together: smoking, bank machine, waiting at bar, physical distance  **If in pub Then**: smoking on drink cycle: 1 smokes other at bar |
| **Rewards:**  **Self: Free:** lush baths, pamper session, film night, time to self/relax, make up  **Self: Cost:** clothes shopping, glamour shoot, comedy show, horse riding  **Group: Free:** pamper, board game night, pj night, film night, come dine with me night, swapping party, beach, picnic, baking night  **Group: Cost**: spa day, bowling, zoo, meal, weekend away |

**Group 0402, session 1**

| **Pros and cons of being drunk**  **Pros**   - Relax - Fun - More sociable - Less inhibited - Find things funnier - To make memories   **Cons**   - Falling - Memory loss - Aggressive - Headache - Hungover - Sick - Tiredness – not a good sleep - Eat less healthy food - Emotionality – teary; giggle - Make risky decisions/actions - Less aware; less safe - Less able to look after self - Mental stability |
| --- |
| **Advantages and disadvantages of not getting drunk during a drinking occasion**  **Advantages**   - Memories: remember night - Safer: putting self at less risk - Safety: sex contact-unintended sex - Handle situations better - Awareness: logical - Healthier - More money - Better sleep - Function better - Better mum – do more with children - Gain mental stability   **Disadvantages**   - Feel less included - Not see humour in things - Not being as sociable - Would see drunk people annoying - Less tolerable |
| **Importance:** 0, 0, 2, 2  **Confidence:** 10, 8 (group), 5  Already changing - children |

**Group 0402 , Session 2**

| 2 bottles of Rose  1 bottle of shots  **IN**  2/3 bottle  1 bottle shots  **OUT**  4 blue WKD  1 cider |
| --- |
| 6.30 – tea  7.30- 9.30 – 2 bottles wine  9.30-00.00 – ½ Malibu  *change mixers from coke to fruit juice  Confidence Importance  Laura 10 10  Amanda 10 9/10  Stef 10 9/10 |
| 1. Single measures 2. Alternate drinks 2 spirits + soft/coke   **Barriers and facilitators to goal attainment**  Barriers  *people buying drinks  *social pressure  Facilitators  *soft drinks  *not to tell others |

**Group 0402, session 3**

| **Risky situations**   - parties – free bar – money is not used as guideline - bereavement – unexpected emotion - celebrations – christenings, weddings - no children - if your partner is there |
| --- |
| Situation: night with no children   - limit amount of money taken - cash only - transfer only have a set amount in account - picking the kids up early - friends group – tell each other - wait for everyone to finish drink - mocktails |
| **Rewards:**  **Cost: self**: cinema, retail, things for the house, body treatment, nails, going out for dinner, sail, snowboarding  **Cost: group**: cinema, weekend away, spa day, beauty day, dinners  **Free: self**: run, baking, x box/games, books, movies  **Free: group**: going to beach, girly night in, movies, pizza, beauty nights, come done with me night, BBQ |

**Group 0403, session 1**

| **Pros and cons of being drunk**  **Pros**   - Confidence - Loss of anxious - Feel dance better - Meet more people - Activities ranged around shots e.g. is drinking <Place name> free drinks student bar - Socialising with casual friends - Adds to the fun. accelerates it, giggling - Dependent on who you are with: more comfortable, less drink   **Cons**   - Tired - Sick - Not able to do things next day - Not able to maintain professional image: “making a tit of self” - Lose inhibitions - Making jokes would not do - Falling over - Throw up - Emotion - Repeating - People reach in different way different group |
| --- |
| **Advantages and disadvantages of not getting drunk during a drinking occasion**  **Advantages**   - Get to pay attention to others funny - Save money - Go to different places - Health benefits - Less hangovers - More with your day   **Disadvantages**   - Less included - Less things to do - Look after everyone. Become burden - Tired - Less comfortable with people you don’t know well - Dealing with drunk people - more stressed - socialising importance - effect of money – more money, more drinks   **Importance:** 1,2,3  **Confidence:** 7,9,7 |

**Group 0403, session 2**

| G meet   - 1. – 3.00 – Coffee   6-9 – 1 bottle of wine  9-9.50; 9.50 – 10.10 – pub/bar – 2 cocktails, 1 beer, 1 shot  10.10 -10.25 – goldslagger  DAD ARRIVES  10.25 – 00.00 – 4 spirits + mixers  00.00 – 1.30 – 2/3 jaeger bombs, ½ VKs |
| --- |
| G’s goal:  Reduce alcohol consumption  Not drink bottle of wine when getting ready + not drinking in the club  WHEN: night out  WHERE: house bottle wine; club  HOW: not buying bottle of drinks; drink water/orange juice instead  WITH: <name>  Importance: 8  Confidence: 8 |
| E’s goal:  No shots in pubs + clubs  WHEN: work night out  WHERE: pubs + clubs  HOW: saying no; pass shot on  WITH: work mates  Importance: 9  Confidence: 9 |
| R:  6-8 – getting ready: ½ bottle of wine or 3 vodkas ( 1 glass)  8.00-12.00 - In house/at part: ½ bottle of wine or 4-6 vodkas  12.00-12.30 - club: chill, 3 sambucas + 5 spirit/mixer  12.30- 02.00 – club: 4 sambucas |
| R’s goal:  Not to have spirit/mixers in clubs  WHEN: on a night out  WHERE: in a club  HOW: replace spirit/mixer with a soft drink or ice  WITH: Gemma/Emma/flat mates  Importance: 6  Confidence: 9 depends if there is an effect |

**Group 0403, session 3**

| **Risky situations:**   - House parties - Own house parties – less expensive (drink more, buy more), jelly shots, no closing time - A closer workmate - People buying you drinks - Someone not seen in a while - Certain people (cousins) - Stressful situations – coursework - Festivals - Paddy’s day - Freshers - Big sporting events - Family events e.g. weddings - Situations get free alcohol - Vulnerable: new situations; break up; bereavement - Holidays: during; after |
| --- |
| If I am at a house party:  If drinking games:  I will:   - Soft drink shots - Comb of alcohol and soft drinks - Faking it - Soft drinks that look like alcohol - Saying no - Drinking less after the game - Avoid drinking games |
| If I am at a house part:  I will:   - Drink from a pint glass - Bring ice - Bring a shot glass to measure alcohol - Avoid free pouring - Alternate soft drinks/water + alcohol: 2 alcohol to 1 soft or bring a bottle of water and have sips |
| **Rewards:**  **Free: Group**: beach/park make most of weather; mocktail night; window shopping; come dine with me; picnics; movie night; pamper session; baking  **Free: Self**: play station; see parents longer; mocktails + strawberries with a book  **Cost: Group**: lunch the next day; cinema + nacho’s; spa; weekend away; fun fair  **Cost: Self**: retail therapy; go to hairdresser’s; get nails done; get tattoo done; spa |

**Group 0501, Session 1**

| **Pros and cons of being drunk**  **Pros:**   - Social aspects - Confidence - Taste - Relax - Have fun - To get out the house - Lose inhibitions - Better dancer   **Cons:**   - Money - Hangovers - Loss of control - Embarrassment - Friends making fun - People’s judgement – strangers, people you know, gender differences - Stigma - Feel old - Lose a day or two - Room spinning – dizzy - Tiring - Look/feel rubbish |
| --- |
| **Advantages and disadvantages of not getting drunk during a drinking occasion**  **Advantages:**   - Health – physical, mental, emotional - More productive next day - Save money   **Disadvantages:**   - Less likely to go out - Feel isolated - Miss out on catching up with people when out |
| **Importance:** 5, 6, 7  **Confidence:** 5, 7, 7 |

**Group 0501, Session 2**

| **SMART**  We will use double measures at home rather than just pouring  How: Use set measuring thimble  **Where:** At home – group + friends’ houses  **When:** Every time drinking together at home  **Whom:** Everyone (group + friends) |
| --- |
| **Barriers and facilitators to goal attainment**  **Barriers:**   - Other friends pouring drinks - Pressure from friends - Speed - Obtaining measuring thimble - Breaking habit – remembering   **Facilitators:**   - Having the thimble - Support from the group - Explaining to other friends the goal - Motivation present to achieve goal |
| **Importance:** 6.5, 6.5, 7  **Confidence:** 6.5, 5, 5 |

**Group 0501, Session 3**

| **High-risk situations**   - Celebrations – distracted, going with flow - Expected/assumption we will drink when get together with friends don’t see too much - Good weather – chillin’ in the back garden – BBQs - General nights out - Going on holiday |
| --- |
| **If – Then**   - If out with friends on special occasions-weddings, then a soft drink in between each alcoholic drink; going to bar to order own drink; dancing - If seeing friends not seen in a while, then as above - If good weather, then mocktails - If general nights out, then alcoholic drinks with less alcohol; taking the car; soft drink in between - If going on holiday, then avoid partying areas; mocktails |
| **Rewards**  **Free:**   - Bubble bath - Early night - Afternoon nap - Reading a book - Pamper night - Walk - Jump on trampoline   **Cost money:**   - Movie night – popcorn etc. - Mocktail night - Cinema - Out for a meal - Pizza in - Going shopping – make-up, clothes - Spa treatment - Ice-skating - Bowling |

**Group 0502, session 1**

| **Pros and cons of being drunk**  **Pros:**   - Social aspects - Becoming more sociable – confidence - Relieves stress - Giddy/carefree - No responsibility – good excuse – “it was the alcohol” - Taste - Bonding   **Cons:**   - Lose control - Losing the next day – tired/hungover - Money - Daft-embarrassing moments - Less cautious – last of safety - Arguments with friends - Overly emotional - Affects memory - Losing inhibitions/too sociable - Health – physical - Psychological/ emotional implications - Addictive - Affects professional standing |
| --- |
| **Advantages and disadvantages of not getting drunk during a drinking occasion**  **Advantages:**   - Health benefits - Not losing a day - Save money - Not becoming dependant on alcohol - Not doing anything silly - ↓embarrassing photos on Facebook - Retaining filter for thoughts - Providing a better role model   **Disadvantages:**   - Reduces social activities - Less tolerant of alcohol levels therefore less able to gauge limit |
| **Importance:** 1, 5, 3  **Confidence:** 8, 6, 4 |

**Group 0502, session 2**

| **Goal:** One Saturday of the month alcohol free catch-up  **How:** make a designated night: last Saturday of the month: drink mocktails as alternative  **Where:** At Leigh-Ann’s house  **When**: Last Saturday of the month  **Who:** All 3 of group |
| --- |
| **Barriers and facilitators to goal attainment**  **Barriers:**   - Peer pressure - In the mood for drinking - Stress - Special occasions   **Facilitators:**   - Appropriate alternative ie. Mocktails - Plan it in advance - Not having any alcohol around - Support from partners - Mindful of parental responsibilities – good role model |
| **Importance:** 10, 10, 7  **Confidence:** 8, 8, 8 |

**Group 0502, session 3**

| **High-risk**   - Boredom - Nights out – pubs etc. - Other people drinking - Being with particular – shots! - BBQs – cider - Out for a meal |
| --- |
| **If-then**   - If bored, then have a bath - If on a night out, then listening to yourself when you’ve hit limit - If other people drinking, then appear to be drinking: always have a drink in front of you - If at BBQs – summer, then limit cool drinks (fridge); having something to eat. - If out for a meal, then a glass of water after every couple of glasses of wine |
| **Rewards:**  **Don’t cost:**   - Have a bath - Bed early to read - Cooking - Meals at friends’ houses - Movie night - Pamper night - Games nights – board games - Spend time with friends - Spend time just yourself   **Cost:**   - Massage - Cooking/baking - New outfit - Make-up - Meet friends for coffee |

**Group 0503, session 1**

| **Pros and cons of being drunk**  **Pros:**   - Social event – getting ready etc. - Relaxing - Confidence - More fun/silly - Lose inhibitions - Shoes don’t hurt when drunk - Meet new people - Ego boost – dress/heels - Look forward to it in the end of the week – reward - Greasy food – guilt free   **Cons:**   - Injuries, bruises - Can’t control emotions – heightened hormones - Money - Hangover - Arguments/fights - Unwanted attention - Excuse for creepy behaviour - Embarrassing moments - Vomiting - Regret sexual misadventures - Lose inhibitions – judgement - Lose the next day – waste time/groggy - Relationship/friendship strain - Greasy food – guilt - Safety-spike drinks - Smoke more - Lose property – bank card etc. |
| --- |
| **Advantages and disadvantages of not getting drunk during a drinking occasion**  **Advantages:**   - Save money - Alternative social activities - Smoke less - Less emotional episodes - Less weight gain - Less injuries - Less creepy attention - Family worry less - Less bad decisions – impulse buying on ebay - Less greasy food - Less fall outs - More stuff done - Health benefits   **Disadvantages:**   - No excuse to buy new dress, lipstick etc. - Feel like missing out on fun nights - Regret not going out - Less of stress release - Wouldn’t see friends as much - Not as many funny stories - Nothing else to do evenings - Tension release with friends - Miss dancing |
| **Confidence:** 10, 10, 5, 6  **Importance:** 5, 0, 6.5, 4 |

**Group 0503, session 2**

| **Goal:** No shots when in a club  **When:** Every club night out  **Where**: In clubs  **How:** Don’t drink it! Tell friends of goal  **With:** All 5 of the group |
| --- |
| **Barriers and facilitators to goal attainment**  **Barriers:**   - Friends buying you shots - Big queue at the bar - Buying a shot with a normal drink - Quicker to drink shots before dance floor - Money – shots are cheaper - £1 easier to pay for - Peer pressure - Miss the buzz   **Facilitators:**   - Team work – supporting each other - Money – buy take away instead - Mocktails instead - More relaxed – talk more - Selective choosing when to go out – not just after SAAS |
| **Confidence:** 5, 10, 7, 10, 1, 10  **Importance:** 5, 0, 10, 5, 5, 5 |

**Group 0503, session 3**

| **High-risk situations**   - Break ups - Everyone going out getting drunk - Failed grade or a good grade - Pay day - Parties – going away party - Concerts/gigs - Grievances, grieving - Holidays - Relationship issues - Long shift - Work - Atmosphere based on alcohol – bars/clubs - Dancing |
| --- |
| **Coping strategies:**   - If break up/fight with bf, then: - Retail therapy; ben & jerry’s; buy food - Movie night with friends - Go for a walk – River side - Failed grade/good grade, then: - go out for a nice meal to celebrate   Phone mum/supportive person   - Payday - Shopping - Savings - Fund for expensive treat - Work, then: - Sleep after - Video games - Picture of boss – dart board - Alcohol atmosphere - Lower % alcoholic drinks ie. Cider or soft drinks - Crisps instead - Pub quizzes, pool, darts |
| **Rewards:**  **Cost money:**   - Dinner with the girls - Take away with girls - Buy clothes - Buy make-up - Date night - Cinema - City trips – Edinburgh - Going home - Dying hair - Cigarettes   **Don’t cost:**   - Bake cakes - Do nails - Do hair - Watch cheesy films - Arts + crafts - Video games – play - Knitting - Get boyfriends to treat us - Sunny – park, beach - People- watching - Walking the dog |

**Group 0504, session 1**

| **Pros and cons of being drunk**  **Pros:**   - Relaxing - Stress relief - Going crazy dancing - Meeting friends - Meeting new people - Fun! – like a high - Carefree – lose inhibitions - Confidence - Feel more open in conversations - Greasy food   **Cons:**   - Share too much - Meeting dodgy people - Vulnerable - Hangover - Nausea/vomiting - Greasy food - Guilt next day - Money - Emotional - High sugar/calories - Tired/lethargic - Health implications - Unproductive next day |
| --- |
| **Advantages and disadvantages of not getting drunk during a drinking occasion**  **Advantages:**   - Remembering everything from night before more clearly - Spending less money - ↓hangover – feel good next day - More control over decisions - Less reckless - More conversation - ↓embarrassed - Better long term health - Observing drunken friends - ↓guilt - ↑productive - Less likely to physically hurt self   **Disadvantages:**   - Having to look after drunk friends - Difficult to find line/peak when drinking. Hard to judge line - Feel less involved - Feel self-conscious of not drinking as much – feel boring |
| **Importance:** 8, 5, 4  **Confidence:** 5, 6, 6 |

**Group 0504, session 2**

| **Goal:**   1. Decant some of your pre-drinking into another bottle and save it for another night 2. Only spend £15-£20 per night out   **When:** Every night out  **Where:** In the flat + in clubs  **How:** Buy container for alcohol + leave bank card at home  **With:** C, A and C |
| --- |
| **Barriers and facilitators to goal attainment**  **Barriers:**   - People around you not taking part in goal – temptation - Special occasions – birthdays etc. - Stress - Feeling upset   **Facilitators:**   - Doing goal together will help you achieve it - Not telling others the goal - Mindful of plans next day |
| **Confidence:** 4, 6, 7.5  **Importance:** 5, 5, 7 |

**Group 0504, session 3**

| **High-risk situations**   - Celebrating - Stress from work (job + uni) - One off occasions (weddings, funerals etc.) - Holidays - Nights out in other cities - Arguments: parents, boyfriends etc. - Disappointment, upset - Bumping into exes - Rounds - Other people drinking lots - Payday |
| --- |
| **If – then:**   - If celebrating, then we’ll keep in mind we want to remember the night; only take £20 out, leave bank card at home - If stress (uni + work), then talk to flatmates about stress; give yourself time to de-stress before a night out (tv, film etc.) - If nights out in other cities, then budget ahead (money for taxi, meals etc.); drinks that are slower to drink (e.g. beer) - If disappointment/feeling down, then crazy dancing in room (like in Girls); listen to “cheer up” playlist; google motivational quotes; create list of own nice quotes (put in jar); ask <name> for a nice quote - Order a glass of water when drinking lots |
| **Rewards:**  **Cost money:**   - Holiday - Get nails done - Hair cut - Clothes shopping - M + S dine for £10 meals - Restaurant - Takeaway - Chocolate   *** any money saved on a night, save in a box that isn’t accessible (padlock) – have money for rewards  **Don’t cost**   - A night to yourself – exfoliating, paint nails - Box set marathon - Beach, parks - Go for a nice walk - Art galleries - Lidl chocolate - Experiment with new recipes - Art work that we want to do e.g. drawings, decoupage, zen drawing - Sit in a café with a book - Go to a book shop e.g. Waterstones - Learn new songs on guitar |

**Group 0505, Session 1**

| **Pros and cons of being drunk**  **Pros:**   - Confidence - Carefree - Lose inhibitions - Social aspect - Meeting new people - Dressing up - High heels - Stress relief   **Cons:**   - Embarrassing - Stupid decisions - Money - Hangovers - Gossip from work mates - Drama/fallouts - High heels - Accidents - Not thinking of the consequences - Unproductive - Messes up diet e.g. greasy food - Sore head - Dehydrated |
| --- |
| **Advantages and disadvantages of not getting drunk during a drinking occasion**  **Advantages:**   - Save money - More time to do things - Less of a “TIT” - Good looks last till the end of the night - No drunken texts - Less likely to damage clothes - Less likely to have accidents   **Disadvantages:**   - Not as many stories to tell/create - Missing out (left out) – pros of drinking - Difficult – less control, peer pressure, buying drinks |
| **Importance:** 4, 5, 5, 4  **Confidence:** 9, 6, 6, 5 |

**Group 0505, session 3**

| **Goal:** only take £10 out to club – <Place name> only  **When**: Every night out exc. Occasions  **Where**: <Place name>  **How:** don’t take bank card out. £10 in purse for going out  **With:** this group of friends |
| --- |
| **Barriers and facilitators to goal attainment**  **Barriers:**   - Take card out - Not getting drunk enough from pre-drinks - Other people buying drinks - Rounds - Birthdays - When pre-drinks isn’t possible - High entry fee - Becca   **Facilitators:**   - Friends striving to goal too - Budget when get paid – plan money for month - Mindful if you stick to it you’ll have a good night - Mindful you’ll have more frequent nights out if save money - Decanting some of bottle into another bottle – limit volume |
| **Importance:** 7, 8, 8  **Confidence:** 5.5, 6, 7.5 |

**Group 0505, session 3**

| **High-risk situations**   - Stress – talk to someone about why stressed before night out - Happy – watch film; road trip - Exams – gym/swimming - Someone’s annoyed me - Having fun/feeling low – tell myself I’m already fun; tell self - wastes money; buy non-alcoholic drinks - Not getting to sleep - A friend feeling down – don’t give in to pressure - Celebrating – some non-alcoholic drinks (pace yourself); tell self no rush to drink – aim should be to celebrate, not get drunk - Disappointment – talk to someone, make yourself feel better e.g. fav film, treat yourself; analyse why disappointed - 1^st^ class train – free wine - Free alcohol - Pub i.e. football |
| --- |
| **Rewards:**  **Cost:**   - New clothes - Going out for dinner - New make-up - Magazines - Food treats e.g. biscuits, mozzarella sticks, flips - Cinema - Sun beds - Road trio - Holiday   **Don’t cost:**   - Movie night - Relaxation night - Pamper night - Make a meal together - Go for a walk |

**Group 0506, Session 1**

| **Pros and cons of being drunk**  **Pros:**   - Open up more - Confidence - Make more friends - Fun - Socialising - Funny memories   **Cons:**   - ↑peeing - Embarrassing moments - Money - Judgement (poor) - Poor decision making - Too much opening up - Safety risks – taken advantage of - Hangover - Unproductive next day - Health negatives - Put on weight – kebabs etc. - Falls, bruises - Drunken texts |
| --- |
| **Advantages and disadvantages of not getting drunk during a drinking occasion**  **Advantages:**   - More money - Remember memories more - No hangovers - Feel healthier   **Disadvantages:**   - Left out of group who are drunk - Feel responsible for drunk friends; look after them - If too sober, wouldn’t feel relaxed enough to meet new people |
| **Importance:** 0, 0, 0  **Confidence:** 8, 8, 10 |

**Group 0506, session 2**

| **Goal:** Use 2 digits (fingers) to measure out vodka/spirits  **When:** if drinking more than once in the week, apply goal to second drinking night; If I’ve got something on the next day  **Where:** pre-drinking in the flat  **How:** as above  **With:** whoever |
| --- |
| **Importance:** 3, 3  **Confidence:** 8, 5 |
| **Barriers and facilitators to goal attainment**  **Barriers:**   - Peer pressure - Not being with other members of the group - Someone else pouring drinks - Drinking other types of alcohol - Harder to stick to when drunk – mark on glass the measure unit; support from group; remind yourself at start of night   **Facilitators:**   - Support from group - Other friends supporting - Not getting drunk - Don’t need any equipment etc. |
| **Group 0506, session 3** |
| **High-risk situations**   - Work - Parties – eat big meal beforehand; slow down drinking; stick to one drink - Family events – don’t compete with family; it’s ok to refuse drink; water in between drinks - Stress - Happy failing tests - Festivals – stay away from fish bowls; have alcohol-free days; limit spending money/night - Holidays - Last-minute plans – go for a meal + have a couple of drinks; buy less alcohol to take to party; don’t steal other people’s drink |
| **Rewards:**  **Cost money:**   - New clothes, band t-shirts - New make-up - Dye hair - Gigs - Cinema - Food treats - Go on holiday - Buy a new book   *** Use money saved from drinking less on this list  **Don’t cost money:**   - A long lie - Move night - A walk to Law Hill - Time to yourself listening to music - Netflix – boxsets - reading |

**Group 0507, session 1**

| **Pros and cons of being drunk**  **Pros:**   - Relaxing - More outgoing/confident - Less self-conscious - Taste - Socialising - Giggle/laugh together - De-stressing - A treat - < inhibitions - >flirty/charming   **Cons:**   - Headache next day - Ill-thought out decisions while drunk - <inhibitions - >flirty - Too honest - Let mouth run away with you - <diplomacy/tact - More emotional when drunk - Hangovers - Vomiting - Room spinning - Plans for next day ruined - Calories from alcohol - Calories from hangover food - Calories from greasy food on night out - More emotional next day - Poor quality sleep – tired next day - Expensive |
| --- |
| **Advantages and disadvantages of not getting drunk during a drinking occasion**  **Advantages:**   - <hangovers - Lose weight - More money - Less impact on health - More productive - Smoke less   **Disadvantages:**   - Wouldn’t enjoy weekend social functions as much - Wouldn’t feel as comfortable around new people - Annoying being around drunk people - Feel like missing out – less involved - Things aren’t as funny - Going home early – keeps you awake when drunk - Eat more |
| **Confidence:** 7.5, 7.5, 6, 6 **Importance:** 3, 3.5, 4, 3 |

**Group 0507, session 2**

| **Goal:** To girls’ night bring 2 bottles of wine (rather than 3) and bring 1 alcohol-free alternative  **When**: 2 out of 4 girls’ nights  **Where**: alternate flats  **How:** organise who brings what before the night  **With**: the awesome crew |
| --- |
| **Barriers and facilitators to goal attainment**  **Barriers:**   - Bad day - Stress - Someone not being fully committed - Other bottles in the house – temptation - Being drunk - False confidence - Sunny day – beer garden   **Facilitators:**   - Group support - Limiting what we’re taking to girls’ night – willpower - Remembering how much better you’ll feel the next morning - Remember you’ll still have fun |
| **Importance:** 5, 5, 5, 6.5  **Confidence:** 6.5, 7, 6, 6 |

**Group 0507, session 3**

| **High-risk**   - Festivals – pre-make, pre-mix bottles (so you’re not mixing when drunk); measure alcohol amount rather than free-pour - Parties – drink own drinks, not communal drinks; alternate with water/juice etc.; make sure eat before - Temptation – alcohol around – don’t buy extra – it’s not around; have goal in mind, encourage/support each other - Birthdays - Bad days - Stress – alternative arranged early – bath/book; treat, something appealing – take away, new top etc, - Celebrations – go for a run - Good news – remind yourself alcohol won’t help with stress - Bad news – rant, talk about it; meditations - Rainy days – sex - Sunny days – achieving something else; baking - Partner wanting to drink - put up barriers to drinking e.g. car; suggest something else to do - Winter – mulled wine – only take small amount of money - Who I’m with – enablers – saying “no”’ plan ahead – anticipate and communicate you’d rather not drink |
| --- |
| **Rewards:**  **Cost:**   - Use calories and money on something else – chocolate, cake - Restaurant - Day out - Massage - Hair cut - Manicure - Shopping - Cinema   **Don’t cost**   - Bath - Favourite programme - Frozen DVD - Give yourself a day off - Day away e.g. hike, drive - Do toe nails |
