# Supplementary material for: Design and feasibility testing of a novel group intervention for young women who binge drink in groups
Source: PLoS One. 2018 Mar 1;13(3):e0193434. doi: 10.1371/journal.pone.0193434 (PMC5832245; doi:10.1371/journal.pone.0193434)
Supplement: S2 Table — (DOCX) [file pone.0193434.s002.docx]

**S2 Table Post study evaluation**

The post-study evaluation forms were completed anonymously. We recorded the group number only

**Questions**

1. What was your main reason for taking part in the study?

2. Was the study different from what you thought it would be? If Yes, how did it differ?

3. Thinking back to the group sessions, what is the first thing that comes to mind?

4. Do you feel you have benefited from the sessions? If Yes, can you try to say in what way? If No, can you try to say why?

5. What techniques have you learned in the study that you would use again in everyday life?

6. Other than the people you are here with, have you discussed the sessions with other women?

7. Would you recommend this study to someone else?

| **Group** | **1 .**  **Reason for taking part?** | **2.**  **Different than expected?** | **3.**  **Memory of intervention sessions?** | **4. Benefited from the sessions?** | **4a.**  **Benefited from the sessions? Comments** | **5.**  **New techniques learned?** | **6. Discuss study?** | **6a. Discussed with whom?** | **7. Recommend study?** |
| --- | --- | --- | --- | --- | --- | --- | --- | --- | --- |
| 0101 | Sounded fun and the chance to win the prize. It was at no inconvenience to us because we got to choose days, times, venues and travel cost was covered | No | We got taught how to make mocktails. They tasted really nice and were a good alternative to alcohol | N | I don’t feel they have changed my opinion on getting drunk but they did give all the facts in a fun, well thought out way | How to make changes in small stages | Y | Friends | Y |
| 0101 | Seemed like fun | Yes. Better than expected but took longer | The activities that were done | Y | Yes. It has made me more aware how often I drink |  | Y |  | Y |
| 0101 | Activities sounded fun and to help with the research | No | Discussing the pros and cons of drinking | Y | Yes. It made me think about women’s attitude to drinking more. Also gave me useful information about setting goals, calories etc. | Making a list of pros and cons. Giving rewards for achieving goals | Y |  | Y |
| 0102 | The fun activities plus getting paid to do it | Not really. I didn’t know what to expect. I expected to be asked about alcohol and I was asked | Entertaining, talking about nights out | Y | Yes, because we had to write down everything we drank. It’s made me more aware | Yes. I am more aware of what I drink now | Y | Friends | Y |
| 0102 | It sounded like a fun way to spend a few hours when we were stressed about fourth year | Not overly | Fun. All of the planned activities were enjoyable and good fun | Y | Yes, It made me consider the downside to drinking as much as I do as often as I did | I found it interesting to fill in a calendar with what I had drunk on each occasion. I would consider doing this in future – if it were an app maybe? | Y | Friends | Y |
| 0102 | Intrigue | Yes. It was very relaxed and not judgmental | Learning about our drinking habits | Y | Yes. Made me more reflective on my drinking | Thinking about how much you drink when you pour your own drinks | N |  | No reply |
| 0104 | A fun thing to take part in with friends at the end of term before everyone left for summer | Yes. It was good at listening to us rather than trying to make us change straight away which could put us off being honest and cooperating | Quite fun | N | Not really. I don’t think my drinking is a problem so I haven’t made a change. I guess some of my friends drinking less could be good! | SMART goals for setting targets | Y |  | Y |
| 0104 | Fun thing to do with my friends | Yes. More focus on the thought processes and perceptions of drinking with young people | It was good fun and beneficial | Y | Yes. Rethink attitude towards drinking and good fun |  | N |  | Y |
| 0104 | Interest + activities sounded fun | Yes. I thought it would be more focus on encouraging us to stop drinking | How interesting it was to hear my friends’ views | Y | Yes. Learned about our group’s attitudes to alcohol | How to make SMART goals. How to reduce alcohol intake if I choose | Y |  | Y |
| 0104 | Thought the activities (mocktail making, make-up etc.) sounded fun | More emphasis on cutting back on drinking than I thought it would be | At first a bit awkward but then really interesting to discuss and got going | Y | Yes. Made me more aware of my drinking and its effects | 1 alcoholic drink and 1 glass of water rule. Stop drinking early (e.g. no drinks after 12 pm). Don’t buy drinks in club (none needed and too expensive) | No reply | Friends | Y |
| 0104 | Interesting topic + fun activities advertised | Yes. I didn’t expect to be given a goal to cut down my drinking | Relaxed sessions that made us open up and really analyse our drinking habits | Y | Yes. I feel confident that if I wanted to I could cut down on my drinking. It also made me realise I am usually more in control than my friends | It is good to stop and evaluate your drinking habits and not think they are just the norm | Y |  | Y |
| 0104 | Thought it would be an interesting experience |  | Interesting to hear everyone’s thoughts on drinking as it is not something that is usually discussed | Y | Yes, whilst I haven’t made any specific goals relating to drinking it has encouraged me to think about it in more detail and I feel like I would be more confident to reduce my drinking in the future if I wanted | Drinking water in between drinks | Y |  | No reply |
| 0201 | Something different to do as a group. Seemed interesting. Wanted the experience – hadn’t done anything like this before. The activities seemed interesting | Yes. Didn’t expect the first session to be in a bar. Much more relaxed than expected. More conversational. Thought it would involve yes/no answers to lots of questions | Setting the goal: Hadn’t heard of SMART goals before. Easy to set big goals but hard to achieve. More appropriate to make small steps. | Y | Yes. Particularly setting goals. Also reflecting on how much you drink. Seeing how silly you can be when drunk | SMART goals | Y | Friends | Y |
| 0201 | Interested to find out what it entailed + to help with medical research | Yes. Really enjoyed the fun tasks, learnt lot about my friends and mine’s behaviours as regards drinking | Being honest about our drinking habits and how it impacts on our lives | Y | Yes. It has made me question what might be described as “expected” behaviour, made me question my drinking behaviour | Setting SMART goals | N |  | Y |
| 0201 | Interest | Yes. It was more fun than I thought it would be. It allured me to spend some quality time with friends | The cocktail/mocktail making night | Y | Yes. I have learned you can definitely enjoy company without alcohol | Making mocktails and thinking about pros vs cons of going out | Y |  | Y |
| 0201 | For a bit of fun, to help out with a study, good karma | Yes. A lot more fun and more relaxed | Just a nice relaxed chilled evening off from studying, but not feeling lazy | Y | Yes absolutely. Definitely follow the goal we set. A lot easier to do when everyone you are out with has the same goal. A bit of peer pressure NOT to do shots now which I never thought would happen | The SMART goal method | Y |  | Y |
| 0202 | Thought it might be interesting and fun | Yes. More of a focus on potentially cutting down alcohol intake | Mocktails and looking at drinking habits | Y | Yes. More aware of alcohol intake and personal consumption | Measuring pre-drinks. Thinking of the cons of being too drunk before drinking too much and considering a more beneficial way to spend the following day to save money | Y |  | Y |
| 0202 | It sounded interesting, a really ear-catching ad. I enjoy doing different things with my evenings and saw it as a chance to get together with the girls | Yes. Much more informal and relaxed. I was surprised we got to choose the time and place rather than being told | The first session comes to mind. We had it in a nice restaurant and I enjoyed seeing the girls and making the mocktails and filling in the calendar | Y | Yes. Even just filling in the last 30 days calendar exercise was such a wake up call that I do drink more often than I realise. It may coincidence but I feel I drink less | I haven’t used it yet but using some kind of visual to count how many drinks I am having could be a good tool | Y | Friends  Family | Y |
| 0202 | Never done anything like that before. Wanted to try something new | Yes. I didn’t think it would be that much fun (mocktails etc.). I really enjoyed it | The first time we listed the alcohol we had (in the calendar) | Y | Yes. I am more aware of what I drink | Thinking more about stuff I wouldn’t have thought about before. For example eating, it’s easy to grab crisps or chocolate. I apply the counting of drinks to food: count how many pieces of healthy and unhealthy food I have a day. I also write down things I can achieve, like a to do list thing, rather than remember them. | Y | Friends  Family | Y |
| 0203 | I thought it be a fun with thing to do with my friends and also a good learning experience | It was so much fun. I thought it be really serious but <name> was very friendly + a good laugh | That is was a good laugh + happy memories | Y | Learned new skills with the classes (mocktails/make up). Helped me look at drinking habits and make changes where needed | Make up techniques are always handy! | Y |  | Y |
| 0203 | Being able to give my point across about not drinking | No | Talking about alcohol and making mocktails | Y | Being able to hear different points of view on alcohol and also give some back |  | Y |  | Y |
| 0204 | To get more info about drinking | No | When I filled in the monthly planner, realised that I was drinking too much | Y | Yes as I have cut out drinking so often | Using the monthly planner | Y |  | Y |
| 0204 | It was interesting | No | The relaxing session | Y | Found out more | Relaxing and make up | Y |  | Y |
| 0204 | To try the courses that we done i.e. make up, massages | No | The different topics | No response |  |  | No reply |  | Y |
| 0204 | To find out more information about drinking | No | How many units are in drinks | Y | As I’m more aware of the units that is in alcohol | How many units are in drink | Y | Friends | Y |
| 0204 | It be fun | No | Can’t remember | Y | Yes. Makes me understand drink more | Drink less | No reply |  | Y |
| 0204 | To get ideas on more things to do with friends that doesn’t involve drink | It was a lot more fun than what I thought it was going to be | Mocktails | Y | Yes. Made me more aware of the effects of binge drinking | Saying no. Finding other ways to socialise and have fun. Thinking healthy. Taking more pride in myself. | No reply |  | Y |
| 0205 | To help out a study of female drinkers of all different types of groups of friends | The study was a lot more fun and brought us all together for something to do and liked speaking about our nights out | We liked speaking about the nights out and I liked making mocktails and the make up session | Y | Yes, I have drank less since my last session | How to save money on nights out. The smokey eye make up | Y | Friends | Y |
| 0205 | To talk more about nights out and to see friends more/socialise. Find out more about how much we drink | No | Fun and interesting | Y | Yes. I have found out and realised how much I have drank on a night out and been surprised | How to measure my alcohol limit, how to cut down/reduce my alcohol intake. How to make goals and plan them | N |  | Y |
| 0205 | I thought it was interesting and sounded fun | No, more fun though | That it was a good laugh and enjoyable, also reminds me to drink less | Y | Yes. I know to control my drinking | Not buy as many drinks | N |  | Y |
| 0206 | Sounded like fun | I did not expect the second two sessions to be goal focussed i.e. about reducing alcohol intake | Mocktails and having a laugh with <name> | Y | It was good to discuss alcohol even just make myself aware of how much I drink | <name>’s wee stress – relaxing facial exercise | Y |  | Y |
| 0206 | My friend found it and we thought it would fun and good thing to take part in. also the thought of winning the spa voucher was tempting | Two of the workshops were not what I expected them to be. The “makeover” part was just something where we learnt how to make the smoky eyes make up and yes it was okay but not what I expected. Also the second part was different | The mocktail session which was definitely the funniest and best part of it! | Y | Yes. Well I started to think about my use of alcohol and thought that perhaps I should reduce it, not to reduce going out but have nights out when I don’t drink and still have a good time. And nights where I drink but not so much that I am completely wasted. | Can’t really think of any specific techniques | N |  | Y |
| 0206 | I thought it was a good idea to help out for the study and also to think about how excessively we drink sometimes | No | The recognition that we were all drinking to excess | Y | Made me realise that I shouldn’t drink as much | Controlling what I drink – trying not to drink excessively | Y | Friends  Family | Y |
| 0206 | Evaluate how much I drink, how it affects me and people around me and hopefully drink less in the future | No | Making lists | Y | Yes. I have been able to reflect on my drinking level and how much it is a social aspect | Setting plans that I can work towards. Small goals that were aiming for a main goal | Y |  | No reply |
| 0206 | Friends thought it would be good idea | Yes, it was more judgemental than I thought | Fun | Y | More aware about alcohol |  | N |  | Y |
| 0207 | Thought it looked fun | No | We had a laugh | N | I still drink as much | n/a | No reply |  | Y |
| 0207 | To see what it was about + for the make up tutorial | No | It was fun | Y | Yes, learned about make up + cocktails | The make up | Y | Family | Y |
| 0207 | It sounded interesting | Yes, I enjoyed the practical things we did | The mocktails! | Y | I have realised how much alcohol I actually consume and I have saw now it effects the tasks I need to do | I learned how to watch the amount of drink I was having | N |  | Y |
| 0208 | Helping out a friend, thought it would be interesting | Didn’t really have any idea what it would be like | We all had very similar ideas in each discussion | Y | Yes. More aware of what I am drinking | Keeping track of what I’m drinking and stopping when I reach a certain amount | N |  | Y |
| 0208 | The make up tutorial and mocktail class sounded good fun, and the money | No | The embarrassment of feeling like I was in an AA meeting | Y | Yes. I’ve realised that it’s important to reward yourself for setting goals and achieving them, and doing something different like a mocktail class with friends can be really good fun | Setting goals and rewarding myself when I achieve them | Y | Friends  Family | N |
| 0208 | To help out a friend | Yes, the enjoyable engaging tasks such as the mocktail making was not expected | Enjoyable, bit of a laugh | Y | Yes, the sessions made me more aware of not only my own but my peers (especially girls) drinking habits. When we discussed what was involved before a night out in the “getting ready” process I was unaware of how much my friends and I actually drank before actually heading out. | Some of the “tactics” we spoke about in avoiding drinks when they are bought for you actually really useful such as, always having two drinks (non alcoholic, no one needs to know) (one in each hand). We also discussed being more aware of what drinks we choose to “pre drink” and limiting that amount and also sharing with a friend.  I often go to the bar alone and order a soft drink that looks like an alcoholic one, this way it stops the peer pressure if your friends are actually aware you are not having an alcoholic drink | Y | Friends  Family | Y |
| 0301 | To see what it was | No | Laughter | Y | Yes. I feel drinking isn’t necessary | Drink isn’t important | Y |  | Y |
| 0302 | To spend time with friends | Yes, I thought we were just getting pampered – I wasn’t told it was a study | Laughing. Making mocktails | Y | I have become more aware of my drinking & setting goals – although this has not stopped me drinking | SMART goals | Y |  | Y |
| 0302 | Fun | Yes, more fun than I thought | Laughing | Y | Yes. More aware of how often I drink | None | N |  | Y |
| 0302 | Spend time with friends | Yes, more interactive tasks. Didn’t realise it would be more than one session | Laughter | N | No. I do not feel any need to change my drinking habits |  | Y |  | Y |
| 0302 | Friends were doing – fun | Yes, didn’t think I’d have as much fun! | Laughing and silliness | N | I’ve stayed the same for drinking habits | The cocktails without alcohol | N |  | Y |
| J0401 |  | Yes, it was more fun and it wasn’t pushing us to stop drinking or making out like we were wrong | We had a good laugh | Y | Yes, I try to be more conscience of my drinking |  | N |  | Y |
| J0401 | I know I drink too much + often and was interested in this | I had no expectations | I felt at ease and enjoyed speaking to my friends about our views on drinking | Y | Yes, I am more aware of my friends’ views and I think about changing my drinking habits more often – I am also more aware how stress affects my drinking | Planning changes to my lifestyle in a specific and manageable manner | N |  | Y |
| 0401 | Seemed like a fun thing to do together | Yes, a lot funner | The talking and questions could maybe be more structured to cut down a bit | maybe | Maybe | Just to think about things more & dangers in everything | Y |  | Y |
| 0402 | Change my drinking habits | No | Activities and realisation of effects of drinking in excess | Y | I changed way I drink and it has made me less tired the day after drinking | Moderation and alternative ways to socialise | Y |  | Y |
| 0402 | Debate + discussion on an important subject | No | Social interaction | Y | Yes. My personal goal. I have improved my low moods. | Making an achievable goal + plan | Y | Friends  Family | Y |
| 0402 | Sounded interesting | Yes. It was a small group so it felt more inclusive |  | Y | I actually drink slightly more than I thought |  | N |  | Y |
| 0402 | Make friends and learn new things | No, it was really interesting and I lots of fun | Fun making the mocktails that was the best | Y | Yes. It shows that you can have fun no alcohol |  | N |  | Y |
| 0403 | Sounded interesting and something fun to do – incentives helped convince me | Yes, very relaxed and casual conversation – not strict as expected; no condemnation when talking about alcohol consumptio | Mocktail making – very fun, gave ideas for alternative ways to drink (without alcohol_ | Y | Yes. Made me more conscious of amount of alcohol I was consuming on nights outs and ways to change it without losing out on the fun | Would consider making mocktails when I want a drink with friends rather than going straight for alcoholic option | Y | Friends | Y |
| 0403 | I think it was a positive study so that people can learn to drink more sensibly and still have fun without any of the negative effects of alcohol | It was so much more thought out, I thought some part would feel more like an experiment | Mocktails because it was so hands on and showed that you could have fun + refreshing drinks without having to drink and can be done with friends | Y | Yes, it has made me see what a lot of people drink more than they need to and how easy it is to reduce it | The mocktail sessions, I feel confident knowing how to use the equipment | Y |  | Y |
| 0203 | It looked interesting | Yes. It was fun, a group of friend having a laugh + chat | Chatting | Y | Yes, it makes me think about how much I drink | The smokey eye make-up | Y |  | Y |
| 0507 | Seemed like fun and my group of friends fit the criteria | Thought it would more about sociability and alcohol – what draws women to it and the psychology instead of cutting down | Interesting | Y | Yes. Think about ways to reduce drinking – like spritz and ways to relax & unwind without a glass of wine | SMART goals. Working in a group for support if feel there is something I want to work on/achieve | Y | Friends | Y |
| 0507 | Thought it would be fun + interesting | It more fun that I thought it would be | Laughing + good reflective chats about drinking + behaviour | Y | Thinking more about the reason I drink + different methods of cutting back | Spritzing our wine with soda to cut down on the wine | Y |  | Y |
| 0507 | Through med student who is in our group taking part | Yes, more interactive and chatty, was expecting more direct questions and answers | Good fun, nice to sit + chat about our different thoughts | N | No. enjoyable + interesting but don’t feel I learned anything, or changed anything I did after | Thinking more about measures of drink when pouring myself. Thinking of other ways to relax /unwind not involving alcohol | Y |  | Y |
| 0507 | The workshop sounded fun | No | Interesting discussions | Y | Yes – I am more aware of triggers and patterns to drinking |  | Y |  | Y |
| 0505 | It sounded interesting | No | Mocktails | Y | I drink less | Weigh up pros and cons about drinking | N |  | Y |
| 0505 | For the mocktail making and the make up artist | Yes, it was impartial to opinion and made me realise that by going out all the time during the semester that it can be damaging. I just thought that the study was research based and it was someone who just wanted to collect information | The goal that we made at the end of the study and whether or not I kept the goal. Which I did! | Y | Yes, I feel that I have realised that limiting what I drink can be beneficial to how I feel about myself. If I don’t drink then I don’t have a horrendous hangover and that I feel I have more control | Making a plan and setting structure and sticking to the plan | N |  | Y |
| 0505 | Because my friend asked me to |  | Having a laugh with everyone | Y | Yes, I understand my drinking pattern much better |  | N |  | Y |
| 0505 | Interesting subject, to help the study | Informal and friendly | Decent chats | Y | Yes, I learnt about myself and my friendship group | Control and support | Y |  | Y |
| 0506 | I thought it would be a good thing to take part in | No | The things we tried as a plan to stop ourselves from drinking so much | Y | Yes. It’s made more aware of the pros of not drinking so much when I’m out with friends | I’ve learned quite a few ways to help me plan to reach different targets | Y |  | Y |
| 0506 | I was interested to learn more | No | Fun and informative | N | No, I already have a controlled view on drinking | Evaluating intake | Y |  | Y |
| 0506 | Something to do after college and gain knowledge | Yes, the girls didn’t judge us for how much we drank | Mocktail lessons! | Y | Yes because it’s made me think about my drinking |  | Y |  | Y |
| 0504 | I thought it would be interesting to pay more attention to how much I drink as I hadn’t done so before | Not really | The pros and cons of drinking | Y | Yes. I feel I have a more conscious way of drinking now rather than being so laid back about it | To measure out drinks. To take less money on a night out. To only take half the amount of alcohol to a party than I usually would | Y | Colleagues | Y |
| 0504 | It sounded interesting and it was something different to take part in | I didn’t really know what to expect – but it turned out to be very enjoyable and we have met some lovely people | It was an eye opener going back over what we drink each weeks | Y | It has made me more aware of the money we spend solely on alcohol and the amount we actually consume | To watch my money spending on solely alcohol. To think more about how much time it takes out of your next being hungover | Y |  | Y |
| 0503 | To help out with the study | Yes, not as as??? I thought it would be | The activities | Y | Yes, closer to friends now | Make-up techniques | Y |  | Y |
| 0503 | Wanting to do activities with my group of friends without drinking | Yes, less formal than I was expecting | Lots of laughing and enjoying the sessions | Y | Yes. I am more aware of how much I drink and the pros and cons of drinking | Being able to talk to my friends when I don’t want to drink & them accepting I’m monitoring how much I drink | Y |  | Y |
| 0503 | Interested, not been in a study before | More fun than I thought | Fun activities and actually thinking about alcohol | Y | I know if I wanted to stop drinking I could | To share worries about alcohol make people reflect on their ways of drinking | Y |  | Y |
| 0503 | Health promotion | No | The activities | Y | More informed about safe measures and more aware of my drinking habits | Unit measurements. Keeping track of how many days I drink | Y |  | Y |
| 0502 | A friend wanted to do it | As expected based on description | The mocktail evening | N | I don’t drink to get drunk or to excess |  | N |  | Y |
| 0502 | I thought it would be interesting, friend asked me | No | Having a giggle with friends | Y | Yes. Previously, although I didn’t drink often, I did drink to excess. Now I consciously choose to drink less | I use the make-up daily, mocktails regularly, goal setting I apply to other areas of my life | Y | Colleagues | No reply |
| 0502 | New experience with friends and to look at how we drink socially | Yes, was a lot more fun and interesting | Making and tasting mocktails | Y | Yes. Benefited our friendship, discussed new activities to do together | Planning ahead, thinking about what I am drinking, making a conscious choice | Y | Friends  Family | Y |
| 0501 | Spending time with friends without alcohol and making a change to drinking habits | No! | Fun, relaxed atmosphere | Y | Yes, made me think more about how much I drink | Mocktails – measuring drinks when pouring at home | Y |  | Y |
| 0501 | To hear about issues surrounding women and alcohol | Yes, it was a lot more fun! | The leader very friendly | Y | Yes. I now drink a smaller amount at home before going out | Smokey eyes make up. Relaxation skills | Y | Colleagues  Family | Y |
| 0501 | Sounded interesting and good fun | No | Making the mocktails | Y | Yes. Made me think about why I drink, the pros and cons and health implications | Measuring of drinks at home using the measure instead of just pouring | Y | Friends  Family | Y |
| 0504 | Boredom! | Was more informal + enjoyable than expected! | <name> (who conducted the sessions) was lovely! Mocktails were really nice! | N | I feel I could set a goal to cut down my drinking if I felt it was necessary. Maybe study would have more of an effect on less strong-willed person! | Rewarding myself when achieving a goal | N |  | No reply |
| 0102 | I thought it would be an interesting study to see how much we drank, also nice to meet up together | Yes. The activities we got to do were more impressive than I thought they would be especially the cocktail one | Nervous to find out the group’s drinking habits | Y | Yes. I knew I didn’t drink as much compared to my friends but looking on the calendar as a whole I am always surprised to see I drink more than I expected | I will definitely write down on a calendar how much I drank on nights out as it shocked me more than I thought it would. It helped me overview my consumption | Y |  | Y |
